# Assessing Life Skills and Adolescent Mental Health: Cross-Cultural Adaptation and Psychometric Evaluation of the Life Skills Scale in Portugal

**DOI:** 10.3390/ejihpe16090121

**Published:** 2026-08-22

**Authors:** Ana Cristina Maia Rocha, Ana João, César Fonseca, Leonel Lusquinhos, Lara Guedes de Pinho

**Affiliations:** 1 NOVA National School of Public Health, Public Health Research Centre, Comprehensive Health Research Centre (CHRC), LA-REAL, CCAL, NOVA University Lisbon, 1600-560 Lisbon, Portugal; 2 University of Évora, 7004-516 Évora, Portugal; 3 School of Health of Santarém, Polytechnic Institute of Santarém, 2005-075 Santarém, Portugal; alsjoao@uevora.pt; 4 Comprehensive Health Research Centre (CHRC), LA-REAL, University of Évora, 7002-554 Évora, Portugal; cfonseca@uevora.pt (C.F.); l.pinho@ess.ipvc.pt (L.G.d.P.); 5 Department of Nursing, University of Évora, 7000-811 Évora, Portugal; 6 Health Sciences Research Unit: Nursing (UICISA: E), Nursing School of Coimbra (ESEnfC), 3046-851 Coimbra, Portugal; oliveira@ese.uminho.pt; 7 School of Nursing, University of Minho, 4710-057 Braga, Portugal; 8 Escola Superior de Saúde, Instituto Politécnico de Viana do Castelo, 4910-023 Viana do Castelo, Portugal

**Keywords:** adolescent, mental health, psychosocial functioning, schools, psychometrics

## Abstract

Life skills are cognitive, emotional and social competencies that help adolescents manage everyday demands and participate in school and social contexts. This study aimed to translate, culturally adapt and examine the psychometric properties of the European Portuguese Life Skills Scale (LSS). A cross-sectional methodological study included a convenience sample of 1807 Portuguese adolescents aged 13–19 years from public secondary schools in Central Portugal. The sample was randomly split for exploratory factor analysis (EFA; *n* = 903) and confirmatory factor analysis (CFA; *n* = 904). EFA supported an interpretable eight-factor solution explaining 50.7% of the variance. CFA compared the original ten-factor model with the EFA-derived eight-factor model. The ten-factor model showed significant standardised loadings (λ = 0.444–0.849) and comparatively better fit, with acceptable root mean square error of approximation (RMSEA = 0.045) and standardised root mean square residual (SRMR = 0.062), although the comparative fit index (CFI = 0.855) and Tucker–Lewis index (TLI = 0.849) remained below preferred thresholds. Internal consistency was satisfactory to excellent (α = 0.782–0.963; ω = 0.783–0.964). Average variance extracted (AVE) and heterotrait–monotrait ratio (HTMT) analyses provided partial evidence of convergent validity and additional support for discriminant validity. Supplementary multi-group CFA provided preliminary support for measurement invariance across female and male adolescents. Findings provide initial support for the European Portuguese LSS as a multidimensional measure of adolescent life skills.

## 1. Introduction

Adolescence is a critical developmental period marked by biological, cognitive, emotional, and social changes (Sawyer et al., 2018; Steinberg & Morris, 2001). During this stage, young people progressively develop autonomy, construct their identity, negotiate peer and family relationships, and respond to increasing academic and social demands (Blakemore & Mills, 2014; Sawyer et al., 2018; Steinberg & Morris, 2001; WHO, 2025). Although these developmental tasks are normative, they may also increase vulnerability to psychological distress, particularly because adolescence overlaps with a developmental window during which many mental disorders first emerge. This vulnerability may be heightened when adolescents have limited resources to manage stress, regulate emotions, communicate effectively, make decisions, solve problems, and cope with everyday challenges (Solmi et al., 2022; Steinberg & Morris, 2001; WHO, 1997, 2020).

Adolescent mental health has become a major public health, educational, and social concern (Johns Hopkins Bloomberg School of Public Health & United Nations Children’s Fund, 2022; United Nations, 2015; WHO, 2025). Depression and anxiety are among the most common mental health difficulties during this developmental period and may negatively affect school engagement, academic achievement, interpersonal relationships, health behaviours, and future well-being (WHO, 2025). Mental health in adolescence should therefore not be understood solely as the absence of symptoms, but as a broader developmental process involving emotional regulation, social functioning, adaptive coping, self-awareness, and the capacity to respond constructively to everyday challenges. Qualitative evidence also suggests that adolescents and young adults describe mental health difficulties in relation to academic pressure, peer and family relationships, social comparison, stigma, and barriers to help-seeking (Hellström & Beckman, 2021). Assessing adolescents’ underlying psychosocial competencies, rather than symptoms alone, may help identify protective resources before difficulties escalate.

Life skills provide a useful framework for understanding and promoting adolescent mental health and well-being. The World Health Organization defines life skills as abilities for adaptive and positive behaviour that enable individuals to deal effectively with the demands and challenges of everyday life (WHO, 1997, 2020). Within this framework, life skills are conceptualised as interrelated cognitive, emotional, and social competencies, including self-awareness, empathy, critical and creative thinking, decision-making, problem-solving, effective communication, interpersonal relationship skills, and coping with emotions and stress. These competencies are directly relevant to adolescents’ ability to understand themselves and others, regulate emotional responses, evaluate situations, make informed decisions, establish positive relationships, and participate effectively in school and social contexts (Darlington-Bernard et al., 2023; Kirchhoff & Keller, 2021; WHO, 1997, 2020).

Recent conceptual and empirical work has reinforced the relevance of life skills and related psychosocial competencies within school health promotion. Decision-making, effective communication, stress management, and problem-solving have been identified as key attributes of life skills that support adolescents’ capacity to cope with health-related challenges, reduce risky behaviours, improve resilience, and strengthen well-being (Torres et al., 2025). Broader reviews similarly suggest that life skills may support emotional regulation, self-awareness, interpersonal communication, problem-solving, and coping strategies (Darlington-Bernard et al., 2023; Kirchhoff & Keller, 2021).

International evidence also supports the value of school-based approaches that target social-emotional and life-skills-related competencies. Social and emotional learning interventions have been associated with improvements in social-emotional skills, attitudes, prosocial behaviour, academic performance, and well-being, as well as reductions in emotional distress and antisocial behaviour (Shi & Cheung, 2024; Taylor et al., 2017). Evidence from universal school-based mental health interventions further suggests that programmes targeting social and emotional processes can benefit children’s and adolescents’ mental health and emotional well-being (Wang et al., 2024). These findings are consistent with positive youth development perspectives, which emphasise young people’s strengths, supportive relationships, and opportunities for meaningful participation in school and community contexts.

Life skills assessment is therefore important for educational, clinical, community, and research purposes, as it may support the identification of adolescents’ psychosocial strengths and difficulties, inform intervention planning, monitor developmental needs and contribute to evaluation of health promotion and mental health prevention programmes (DeVellis & Thorpe, 2022; International Test Commission, 2017; WHO, 1997, 2020). Identifying adolescents’ strengths and difficulties across different life skill domains can help teachers, school health professionals, nurses, psychologists, youth workers, and researchers design, monitor, and evaluate interventions tailored to young people’s developmental needs. Psychometrically sound instruments are also needed to examine how life skills relate to mental health indicators, such as anxiety and depressive symptoms, and to determine whether life skills programmes are effectively addressing the competencies they aim to develop.

Among the available instruments, the Life Skills Scale (LSS), originally developed in Turkey by Erduran Avcı and Korur (2022), offers a comprehensive measure of adolescents’ general life skills. The original Turkish version comprises 83 items distributed across ten subdimensions: critical thinking, creative thinking, decision-making and problem-solving, coping with stress and emotions, interpersonal relationships and communication, empathy, self-awareness, self-esteem, teamwork, and social responsibility. In the original validation study, exploratory and confirmatory factor analyses supported the multidimensional structure of the scale, and reliability analyses provided evidence of adequate internal consistency (Erduran Avcı & Korur, 2022).

However, the use of a scale developed in one linguistic and cultural context requires careful cross-cultural adaptation before it can be applied in another population. Although life skills refer to broadly relevant psychosocial competencies, their expression, interpretation, and perceived importance may vary across cultural, educational, and social contexts. Differences in language, school experiences, communication norms, family expectations, adolescent social roles, and youth service provision may influence how young people understand and respond to scale items, reinforcing the need for rigorous cross-cultural adaptation and psychometric evaluation before using an instrument in a new population (Beaton et al., 2000; International Test Commission, 2017). International guidelines therefore recommend rigorous translation and adaptation procedures to ensure semantic, idiomatic, conceptual, and cultural equivalence between original and adapted versions of an instrument (International Test Commission, 2017).

Despite the relevance of life skills for adolescent mental health, educational development, school health promotion, and youth support services, no culturally adapted and psychometrically evaluated European Portuguese version of the LSS is currently available for Portuguese adolescents. This represents an important gap for research and practice, as the absence of culturally appropriate instruments limits the ability to identify adolescents’ life skills, examine their association with mental health indicators, and design school-based or community-based interventions tailored to young people’s needs.

Therefore, this study aimed to translate, culturally adapt, and evaluate the psychometric properties of the European Portuguese version of the LSS in a school-based sample of Portuguese adolescents. Specifically, it examined the scale’s internal structure using exploratory factor analysis (EFA) and confirmatory factor analysis (CFA) in independent subsamples, assessed reliability, and explored evidence of convergent and divergent validity through correlations among LSS dimensions and with depressive and anxiety symptoms. By providing a culturally adapted and psychometrically evaluated instrument, this study may support the assessment of adolescent life skills and inform school- and community-based practices aimed at promoting mental health, psychosocial competence, and well-being.

## 2. Materials and Methods

### 2.1. Study Design

This quantitative, cross-sectional methodological study evaluated the psychometric properties of the European Portuguese version of the LSS in a school-based sample of Portuguese adolescents. As this was a non-experimental validation study, no variables were manipulated. Each participant completed the assessment once, although recruitment and data collection occurred across two academic years: 2024–2025 and 2025–2026. The study assessed adolescents’ life skills, sociodemographic characteristics, and mental health indicators, including anxiety and depressive symptoms.

### 2.2. Participants and Procedures

A convenience sample of 1807 adolescents aged 13–19 years was recruited from public secondary schools located in the Central region of Portugal during the 2024–2025 and 2025–2026 academic years. Inclusion criteria were enrolment in public secondary education, fluency in Portuguese, and the ability to independently understand and complete the questionnaires. Adolescents with diagnosed cognitive impairments that could hinder comprehension of the questionnaire items were excluded. This criterion was verified by the class teacher, who identified students whose known cognitive difficulties or educational records indicated that they might be unable to understand or complete the instruments autonomously.

Although no formal a priori power analysis was conducted, the adequacy of sample size was evaluated according to methodological recommendations for psychometric studies and factor analysis (Brown, 2015; Hair et al., 2019). The final sample included 1807 adolescents for an 83-item scale, corresponding to approximately 21.8 participants per item. After random split-sample validation, the EFA and CFA subsamples included 903 and 904 participants, respectively, corresponding to approximately 10.9 participants per item in each subsample. These values exceed commonly cited recommendations for factor analysis, which suggest minimum subject-to-item ratios of 5:1 to 10:1 and/or absolute sample sizes of at least 300 participants (Hair et al., 2019). Therefore, the sample size was considered adequate for the planned exploratory and confirmatory analyses.

Participation was voluntary. Informed consent was obtained from participants aged 18 years or older. Among adolescent participants who were minors, informed assent was obtained from the adolescents and informed consent was obtained from their legal guardians before participation. Only students for whom the required consent/assent procedures had been completed were given access to the online questionnaire. The accessible population comprised 3247 eligible students. Of these, 1807 adolescents participated in the study, corresponding to an overall participation rate of 55.7%. Records distinguishing between students who did not participate because parental or legal guardian consent was not provided and those who did not provide assent were maintained locally by the class teachers and schools, in accordance with school procedures and confidentiality requirements. Consequently, these two sources of non-participation could not be quantified separately by the research team.

Data collection took place after formal authorisation was obtained from the Directorate-General for Education through the School Survey Monitoring Platform, as well as from the school management boards. The questionnaires were completed anonymously during class time, under teacher supervision, using a secure online platform. Students completed the survey using their personal mobile phones. The average completion time was approximately 15–20 min. Students who did not participate remained under teacher supervision and completed the alternative activity defined by the teacher, in accordance with usual classroom procedures.

No financial or material compensation was provided to participants. The absence of incentives was considered appropriate given the voluntary nature of participation, the minimal burden of completing the questionnaire, and the context of data collection within regular school activities.

### 2.3. Instruments

#### 2.3.1. Sociodemographic Questionnaire

The sociodemographic questionnaire collected information on age, sex assigned at birth, gender identity, school grade, academic performance, parental education, school-based social support level, and extracurricular activities.

#### 2.3.2. Life Skills Scale

The LSS (Erduran Avcı & Korur, 2022) is an 83-item self-report instrument designed to assess adolescents’ general life skills. The original Turkish version comprises ten subdimensions: critical thinking, creative thinking, decision-making and problem-solving, coping with stress and emotions, interpersonal relationships and communication, empathy, self-awareness, self-esteem, teamwork, and social responsibility. Items are rated on a five-point Likert-type scale ranging from 1 (“totally disagree”) to 5 (“totally agree”). Higher scores indicate higher perceived life skills, except for the negatively worded Teamwork items, which require interpretation according to item direction.

In the original validation study, exploratory and confirmatory factor analyses supported the ten-factor structure of the LSS. The ten-factor solution explained 51.07% of the total variance, and item factor loadings in the exploratory factor analysis ranged from 0.326 to 0.805. CFA further supported the adequacy of the ten-factor model, with fit indices within acceptable ranges. The original study also reported high internal consistency for the overall scale and adequate reliability across the ten subdimensions (Erduran Avcı & Korur, 2022).

In the present study, the European Portuguese version of the LSS was used following translation and cultural adaptation procedures. Evidence based on internal structure and relations with other variables is reported in the Results Section.

#### 2.3.3. Patient Health Questionnaire-9 for Adolescents

The Patient Health Questionnaire-9 for Adolescents (PHQ-9) (Johnson et al., 2002) is a nine-item self-report scale that assesses depressive symptoms during the previous two weeks. Items are rated from 0 (“not at all”) to 3 (“nearly every day”), with total scores ranging from 0 to 27. Higher scores indicate greater depressive symptom severity. The PHQ-9 has demonstrated good reliability and validity in adolescent populations (Andreas & Brunborg, 2017; Anum et al., 2019; Fonseca-Pedrero et al., 2023; Ganguly et al., 2013; Tsai et al., 2014).

#### 2.3.4. Generalized Anxiety Disorder-7

The Generalized Anxiety Disorder-7 (GAD-7) (Spitzer et al., 2006) is a seven-item self-report instrument that assesses anxiety symptoms during the previous two weeks. Items are rated on a four-point scale ranging from 0 (“not at all”) to 3 (“nearly every day”), with higher scores indicating greater anxiety symptom severity. The Portuguese adolescent version has shown excellent internal consistency, with Cronbach’s α = 0.93, and has been used in clinical and community samples (Gonçalves & Santiago, 2019).

### 2.4. Translation and Cultural Adaptation

The LSS was translated and culturally adapted from the original Turkish version into European Portuguese in accordance with the International Test Commission Guidelines for Translating and Adapting Tests (International Test Commission, 2017). This process was undertaken to ensure semantic, idiomatic, conceptual, and cultural equivalence between the original instrument and the European Portuguese version, as linguistic and cultural differences may influence adolescents’ comprehension, interpretation, and response processes to items.

The adaptation process was conducted in several sequential stages. First, two bilingual translators independently translated the instrument from Turkish into European Portuguese. Translators were instructed to focus not only on literal translation but also on semantic equivalence, conceptual accuracy, linguistic naturalness, and suitability for adolescents in the Portuguese cultural context. The two translated versions were then compared item by item, and discrepancies related to wording, terminology, or cultural adequacy were discussed until a single consensus version was produced.

Second, two independent translators, who had not participated in the initial translation stage and were blind to the original Turkish version, back-translated the consensus European Portuguese version into Turkish. The back-translated versions were compared with the original instrument to identify possible shifts in meaning, conceptual inconsistencies, or loss of nuance. The back-translated versions were subsequently reviewed by the original authors of the LSS, who assessed their content equivalence and confirmed that the adapted version adequately preserved the original instrument’s meaning and conceptual content. Any discrepancies identified during this stage were reviewed by the research team, leading to further refinement of the preliminary European Portuguese version.

Third, an expert panel reviewed the preliminary European Portuguese version. The panel consisted of five members: one psychologist, one psychiatrist, two nurses, and one linguist. Of the two nurses, one was specialised in mental health and psychiatric nursing, and the other in child health and paediatric nursing. The experts evaluated each item for semantic equivalence, conceptual relevance, clarity, cultural appropriateness, and adequacy for the target age group. Suggested modifications were discussed collectively, and item wording was revised as necessary to improve comprehensibility and cultural fit while preserving the original instrument’s meaning. During this stage, items 9, 25, 33, and 57 were identified as requiring linguistic simplification to improve clarity and age-appropriateness for Portuguese adolescents. These adjustments involved simplifying sentence structure, avoiding potentially ambiguous wording, and using clearer European Portuguese phrasing, while preserving the original meaning and intended life skills domain. Finally, the pre-final version was pilot-tested with 30 adolescents from the target population. This stage aimed to assess the clarity, comprehensibility, and perceived relevance of the items and response format. The research team reviewed feedback from the adolescents, and minor wording refinements were introduced to enhance linguistic naturalness and ensure that the final European Portuguese version was culturally appropriate and easily understood by adolescents.

### 2.5. Statistical Analysis

Data analyses were performed using IBM SPSS Statistics, Version 29.0 (IBM Corp., 2023), and JASP, Version 0.95.4 (JASP Team, 2025). To minimise capitalisation on chance and allow cross-validation of the factor structure, the total sample (*N* = 1807) was randomly divided into two independent subsamples. The first subsample was used for exploratory factor analysis (EFA; *n* = 903), and the second subsample was used for confirmatory factor analysis (CFA; *n* = 904). Given the five-point Likert-type response format of the LSS items and the large sample size, item scores were treated as approximately continuous in the EFA and CFA. This decision was made to allow comparison with the original validation study and to support the estimation of complex multidimensional models. The ordinal nature of the response format was considered when interpreting the findings, and future studies should examine the robustness of the factor structure using polychoric correlation matrices and ordinal estimators.

### 2.6. Preliminary Analyses

Descriptive statistics were computed in the full study sample (*N* = 1807) to characterise participants and examine item distributions. Frequencies and percentages were calculated for categorical variables, whereas means and standard deviations were calculated for continuous variables. Percentages were reported together with absolute frequencies whenever applicable. The suitability of the data for factor analysis was assessed using the Kaiser–Meyer–Olkin (KMO) measure of sampling adequacy and Bartlett’s test of sphericity.

Missing data were examined before analysis. Percentages for sociodemographic variables were calculated using valid responses, as the number of missing responses varied across variables. No imputation of missing data was performed. Analyses in JASP were conducted using the available valid data for each analysis; therefore, the number of valid cases could vary across variables or pairs of variables. The valid sample size is reported for each main psychometric analysis.

### 2.7. Exploratory Factor Analysis

EFA was conducted in the first randomly selected subsample to examine the underlying dimensionality of the European Portuguese version of the LSS. Principal axis factoring was used as the extraction method, as the aim was to identify latent constructs underlying adolescents’ responses to the scale items rather than to perform data reduction. An orthogonal Varimax rotation was applied to facilitate factor interpretation, improve the clarity of item groupings, and allow comparability with the original validation study of the LSS. Although life skill domains are theoretically interrelated and oblique rotations are often appropriate when factors are expected to correlate, Varimax rotation was retained because it produced the clearest and most theoretically interpretable exploratory solution and enabled comparison with the original scale development study. The interrelations among LSS dimensions were subsequently examined through Spearman’s correlations, and the potential need for oblique or more flexible modelling approaches is addressed in the Discussion and Future Research Sections.

Factor retention was based on a combination of statistical and conceptual criteria, including eigenvalues greater than 1, inspection of the scree plot, the percentage of explained variance, and the theoretical interpretability of the extracted solution, following commonly used recommendations for exploratory factor analysis (Hair et al., 2019). Factor loadings ≥ 0.40 were considered meaningful for item interpretation, whereas lower loadings were generally not interpreted unless they were theoretically relevant to understanding the structure of the scale (Hair et al., 2019). The conceptual coherence of each factor, the presence of cross-loadings, and item uniqueness values were also considered when evaluating the factor solution. This analytic strategy was broadly consistent with the original validation study of the LSS, which also used an exploratory approach with Varimax rotation and considered eigenvalues, explained variance, factor loadings, and theoretical interpretability when establishing the scale structure (Erduran Avcı & Korur, 2022).

### 2.8. Confirmatory Factor Analysis

CFA was conducted in the second randomly selected subsample to examine the adequacy of the factor structure of the European Portuguese version of the LSS. Two models were tested: the original theoretical ten-factor model, comprising 83 items, and the alternative eight-factor model derived from the EFA, comprising 78 items. The latter included items that loaded meaningfully on the exploratory factors, excluding those that did not meet the predefined loading criterion in the rotated EFA solution.

The robust maximum likelihood (MLR) estimator was used. This estimator was selected because of the large sample size and the five-point response format of the LSS items, and because it provides robust standard errors and fit indices corrected for potential violations of multivariate normality.

Model fit was evaluated using the chi-square statistic (χ^2^), the comparative fit index (CFI), the Tucker–Lewis index (TLI), the root mean square error of approximation (RMSEA), and the standardised root mean square residual (SRMR). Additional indices, including the non-normed fit index (NNFI), incremental fit index (IFI), relative noncentrality index (RNI), parsimony normed fit index (PNFI), goodness-of-fit index (GFI), and expected cross-validation index (ECVI), were also reported to provide complementary information about model performance and model comparison. Lower ECVI values were interpreted as indicating comparatively better fit among competing models.

Model fit was interpreted using a combined evaluation of fit indices, standardised factor loadings, theoretical interpretability, and parsimony, rather than relying on a single cut-off value. In line with commonly used recommendations for applied CFA, CFI and TLI values closer to 0.95, RMSEA values ≤ 0.08, and SRMR values ≤ 0.08 were considered indicative of acceptable to good fit, while also taking into account model complexity and the theoretical adequacy of the factor structure (Brown, 2015).

### 2.9. Reliability and Validity Evidence

Internal consistency was assessed using Cronbach’s alpha and McDonald’s omega for the total LSS and for each of the ten dimensions retained as the primary scoring framework. Values of α and ω ≥ 0.70 were considered satisfactory, following commonly used recommendations for scale reliability (DeVellis & Thorpe, 2022). The Teamwork dimension comprises negatively worded items; therefore, scores for this dimension were interpreted according to item direction.

Convergent and discriminant validity at the measurement model level were further examined using average variance extracted (AVE) and the heterotrait–monotrait ratio (HTMT), respectively. AVE values close to or above 0.50 were considered supportive of convergent validity, particularly when accompanied by satisfactory reliability estimates. HTMT values below 0.85 were interpreted as supporting discriminant validity among the retained dimensions.

Validity evidence based on relations with other variables was examined through correlations between LSS dimensions and mental health indicators, namely depressive symptoms assessed with the PHQ-9 and anxiety symptoms assessed with the GAD-7. Given the ordinal response format of the LSS items and the possibility of non-normal score distributions, Spearman’s rank-order correlations were used. Negative associations between most LSS dimensions and symptoms of anxiety and depression were expected, particularly for dimensions related to emotional regulation, self-awareness, and self-esteem. Statistical significance was set at *p* < 0.05.

### 2.10. Supplementary Measurement Invariance Analysis

As a supplementary analysis, measurement invariance across sex was examined using multi-group confirmatory factor analysis among female and male adolescents. The LSS items were treated as approximately continuous indicators, and the measurement invariance models were estimated using maximum likelihood (ML). A sequence of increasingly constrained models was evaluated. The configural model tested whether the same factorial structure was supported across female and male adolescents without imposing equality constraints on model parameters. Metric invariance was subsequently examined by constraining factor loadings to equality across groups. Scalar invariance was then tested by retaining the equality constraints on factor loadings and additionally constraining item intercepts to equality across groups. Measurement invariance was evaluated by examining changes in CFI, RMSEA, and SRMR between successive models, with emphasis on whether the additional equality constraints resulted in a meaningful deterioration in model fit.

### 2.11. Ethical Considerations

This study was approved by the ethics committee on 13 January 2025 (approval no. 24064) and authorised by the national educational authority through the School Survey Monitoring Platform on 2 February 2025. The study was conducted in accordance with the principles of the Declaration of Helsinki and applicable data protection regulations. Participation was voluntary, anonymous, and without financial or material compensation. Participants were informed about the aims of the study, the nature of their participation, the approximate time required to complete the questionnaires, and their right to decline participation or withdraw before submitting the questionnaire, without any penalty or consequence.

Informed consent was obtained from participants aged 18 years or older. Among adolescent participants who were minors, informed assent was obtained from the adolescents and informed consent was obtained from their legal guardians before participation. Data were collected anonymously through a secure online platform during class time, under teacher supervision. No personally identifiable information was collected, and individual responses could not be linked to participants after submission.

All data were stored on password-protected institutional servers. Access to the anonymous dataset was restricted to authorised members of the research team involved in data analysis and interpretation. Data were handled in accordance with applicable data protection requirements and will be securely retained for five years following publication, after which they will be permanently deleted.

## 3. Results

Overall, the European Portuguese version of the LSS showed satisfactory psychometric performance in this adolescent sample. The EFA supported a multidimensional structure, although with some reorganisation of the original domains. The CFA indicated that the original ten-factor model performed better than the alternative eight-factor solution, although support for the model was partial rather than definitive, with acceptable RMSEA and SRMR but incremental fit indices below conventional thresholds. Supplementary measurement invariance analysis provided preliminary support for configural, metric, and scalar invariance across female and male adolescents. Internal consistency was satisfactory to excellent across the total scale and dimensions. Additional analyses provided partial evidence of convergent validity and support for discriminant validity, and associations with PHQ-9 and GAD-7 scores supported theoretically coherent links between emotional and self-related life skills and symptoms of depression and anxiety.

### 3.1. Sample Characteristics

The sample comprised 1807 secondary school students. Most participants were aged between 15 and 17 years, with 16-year-olds representing the largest group (31.8% of valid responses), followed by 17-year-olds (30.6%) and 15-year-olds (27.0%).

Regarding nationality, most students were Portuguese (*n* = 1640, 91.1%), while Brazilian students constituted the largest foreign-nationality group in the sample (*n* = 110, 6.1%). Participants of other nationalities collectively represented 2.8% of valid responses (*n* = 51).

With respect to sex assigned at birth, 58.3% of participants reported being female (*n* = 1049), 39.6% male (*n* = 713), 0.6% intersex (*n* = 11), and 1.5% preferred not to answer (*n* = 27). Regarding gender identity, most participants identified as cisgender (*n* = 1653, 93.3%). Transgender participants represented 1.2% of valid responses (*n* = 21), non-binary participants 0.9% (*n* = 16), and 4.6% preferred not to answer (*n* = 81).

By school year, 41.6% of participants were in the 10th grade (*n* = 737), 30.7% in the 11th grade (*n* = 543), and 27.7% in the 12th grade (*n* = 491).

Students’ perceived academic achievement was generally positive: 74.4% rated their achievement as good or very good (*n* = 1315), whereas only 2.3% rated it as poor or very poor (*n* = 41). Similarly, satisfaction with academic performance was favourable, with 63.5% of students reporting being satisfied or very satisfied (*n* = 1124). By contrast, only 9.1% reported being dissatisfied or very dissatisfied (*n* = 162).

Regarding family background, 33.7% of mothers (*n* = 597) and 23.7% of fathers (*n* = 419) had completed higher education. Secondary education was the most frequent educational level among both mothers (*n* = 532, 30.1%) and fathers (*n* = 498, 28.2%).

With respect to socio-economic status, as measured by the Portuguese School Social Support scheme, most students did not receive any level of support (*n* = 1163, 65.9%). Among beneficiaries, level C was the most frequent category (*n* = 248, 14.1%), followed by level B (*n* = 236, 13.4%) and level A (*n* = 117, 6.6%).

Finally, a substantial proportion of students reported participating in extracurricular activities, with 63.1% (*n* = 1113) indicating involvement (Table 1).

### 3.2. Sample Adequacy for Exploratory Factor Analysis

The suitability of the data for EFA was examined in the first randomly selected subsample (*n* = 903). The overall KMO value was 0.963, indicating excellent sampling adequacy. Item-level measures of sampling adequacy ranged from 0.835 to 0.982, showing that all items met the recommended criteria and shared sufficient common variance for factor analysis. Bartlett’s test of sphericity was statistically significant, χ^2^(3403) = 46,021.915, *p* < 0.001, indicating that the correlation matrix differed significantly from an identity matrix and confirming the appropriateness of factor analysis. A chi-square goodness-of-fit test associated with the extracted factor solution was also statistically significant, χ^2^(2767) = 7035.906, *p* < 0.001. Given the large sample size and the well-documented sensitivity of chi-square statistics to minor residual discrepancies, this result was interpreted with caution and was not considered in isolation when evaluating the adequacy of the factor solution. Instead, the overall assessment was based on multiple complementary criteria, including the KMO measure, Bartlett’s test, eigenvalues, the Scree plot, factor loadings, explained variance, item uniqueness values, and the theoretical interpretability of the extracted factors. Together, these findings supported the factorability of the LSS and the suitability of conducting EFA in the Portuguese adolescent sample.

### 3.3. Exploratory Factor Structure of the LSS

Principal axis factoring with Varimax orthogonal rotation was performed to obtain a simple, parsimonious, and readily interpretable factor structure. Varimax rotation was selected because the original LSS was developed as a multidimensional instrument comprising ten conceptually distinct life skill domains. This rotation method maximises the variance of factor loadings across factors, thereby facilitating the identification and interpretation of the underlying dimensions while maintaining consistency with the original factorial structure. Although oblique rotation methods permit correlations among factors, they were not adopted because the primary objective of this exploratory analysis was to derive a clear and parsimonious factor solution that could be directly compared with the original version of the scale.

**Figure 1:**
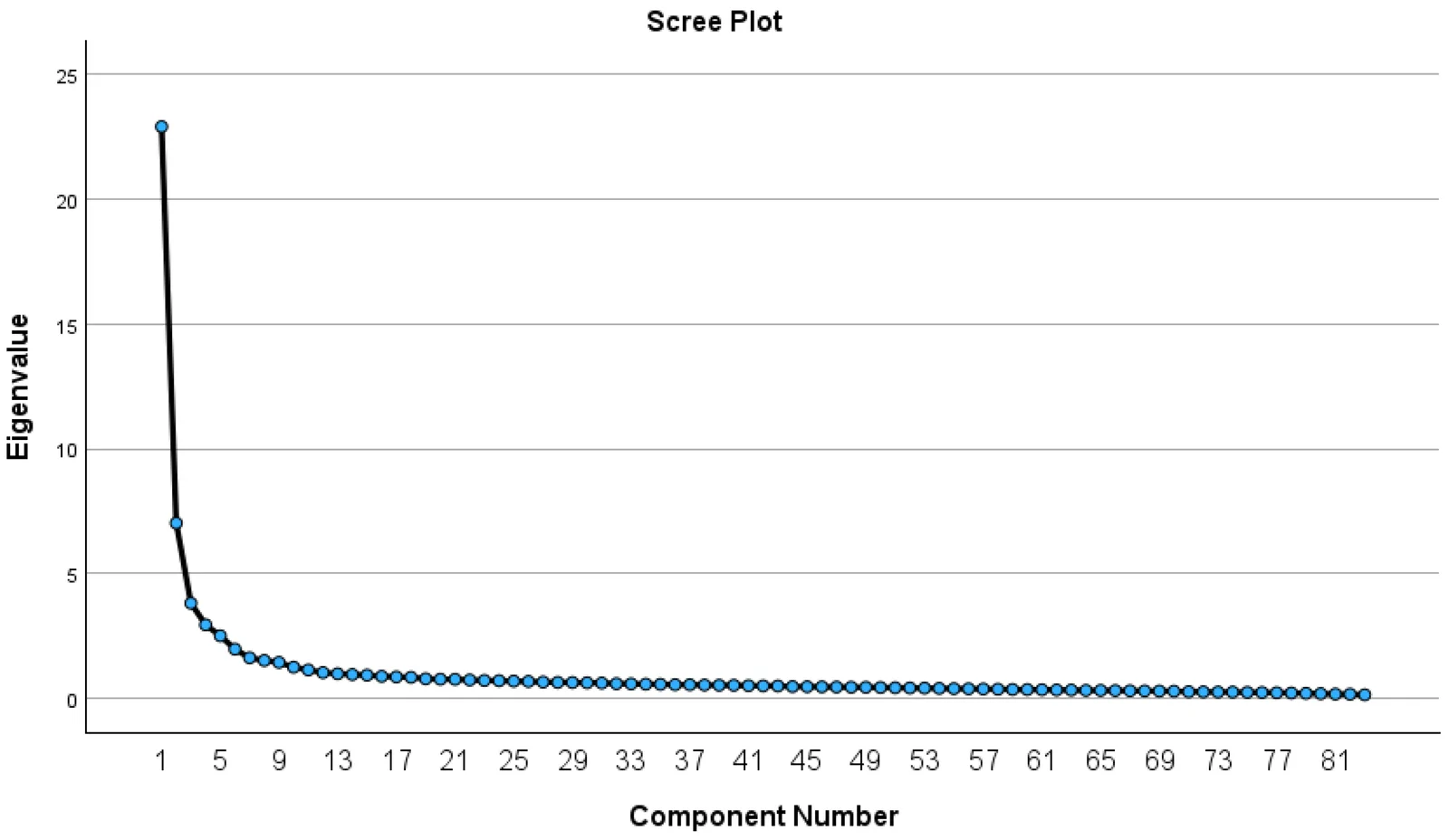
Scree plot of the exploratory factor analysis of the European Portuguese version of the Life Skills Scale.

Factor retention was based on the combined consideration of initial eigenvalues greater than 1, inspection of the Scree plot, explained variance, factor loadings, item uniqueness values, and theoretical interpretability. The Scree plot (Figure 1) showed a marked decline in eigenvalues across the first components, followed by a gradual levelling-off of the curve, supporting the presence of a multidimensional structure. The analysis suggested an interpretable eight-factor solution for the European Portuguese version of the LSS. The retained factors had initial eigenvalues greater than 1 and together explained 50.7% of the total variance (Table 2).

Although the exploratory solution did not fully reproduce the original ten-dimensional structure of the LSS, the extracted factors were broadly consistent with the instrument’s conceptual framework and reflected meaningful cognitive, emotional, interpersonal, self-evaluative, and prosocial life-skills domains.

In the rotated solution, Factor 1 accounted for 12.0% of the variance and predominantly included items from the Decision-Making and Problem-Solving and Critical Thinking dimensions. Factor 2 accounted for 8.7% of the variance and consisted mainly of Self-Esteem items, along with some Self-Awareness items. Factor 3 accounted for 6.3% of the variance and grouped items from Coping with Stress and Emotions. Factor 4 accounted for 6.1% of the variance and mainly represented Self-Awareness. Factor 5 accounted for 5.8% of the variance and included items from Social Responsibility and Empathy. Factor 6 accounted for 5.0% of the variance and corresponded primarily to Creative Thinking. Factor 7 accounted for 4.1% of the variance and represented Teamwork. Finally, Factor 8 accounted for 2.8% of the variance and partially comprised items from Interpersonal Relationships and Communication.

The initial eigenvalues of the eight retained factors ranged from 23.63 for Factor 1 to 1.05 for Factor 8. Overall, the exploratory solution indicated a multidimensional organisation of the European Portuguese LSS, while also showing partial empirical overlap across some theoretically distinct domains. This overlap was particularly evident for Decision-Making and Problem-Solving with Critical Thinking, Self-Esteem with Self-Awareness, and Social Responsibility with Empathy. These patterns are further interpreted in the Discussion section. The item-level loading pattern is described in the following section.

### 3.4. Factor Loadings and Uniqueness

Analysis of the rotated factor solution showed that most items presented meaningful loadings, generally exceeding the predefined criterion of λ ≥ 0.40. Salient factor loadings, including cross-loadings ≥ 0.40, and uniqueness values are provided in Supplementary Table S1. The strongest loading within Factor 1 was observed for item 23, “I use my experience to solve problems and make important decisions” (λ = 0.656), while the highest loading in Factor 2 was observed for item 70, “I believe I can do many things” (λ = 0.780). The highest loadings for the remaining factors were observed for item 35 in Coping with Stress and Emotions (λ = 0.617), item 56 in Self-Awareness (λ = 0.703), item 81 in Social Responsibility and Empathy (λ = 0.772), item 11 in Creative Thinking (λ = 0.701), item 76 in Teamwork (λ = 0.797), and item 42 in Interpersonal Relationships and Communication (λ = 0.543).

Uniqueness values were generally moderate, suggesting that most items were adequately represented by the extracted factors. The lowest uniqueness values were observed for Self-Esteem items, particularly items 70, 66, 69, and 68, indicating that a substantial proportion of their variance was explained by the factor solution. By contrast, higher uniqueness values were observed for items 1, 30, 5, 33, 80, and 29, suggesting that these items were less strongly explained by the extracted factors.

A small number of items did not reach the conventional loading threshold in the rotated solution, namely items 1, 41, 44, 45, and 50. These items were retained at this stage because of their theoretical relevance and contribution to the content coverage of the original LSS, but they should be examined more closely in future studies. Overall, the item-level results suggested that most items contributed adequately to the exploratory structure of the European Portuguese LSS, while indicating that some items may require further psychometric evaluation.

### 3.5. Descriptive Statistics for LSS Items and Dimensions

Item-level descriptive statistics are presented in Supplementary Table S2. Overall, participants reported moderate to high levels of perceived life skills, with most item means exceeding the midpoint of the response scale. The highest item scores were observed for statements related to prosocial behaviour, empathy, responsibility towards others, and reflective decision-making. By contrast, lower item scores were observed for statements related to anxiety, positive thinking, perfectionism, and perceived self-worth, suggesting comparatively greater perceived difficulties in emotional regulation, stress management, and self-evaluation among some adolescents.

The negatively worded Teamwork items showed comparatively low mean scores. Considering the direction of these items, this pattern indicates generally favourable attitudes towards collaboration, shared responsibility, and acceptance of different perspectives in group contexts.

Dimension-level descriptive statistics, computed according to the original ten-dimensional scoring structure of the LSS, are presented in Table 3.

Mean scores ranged from 2.42 to 4.24 on a 1–5 response scale. The highest mean score was observed for Empathy (*M* = 4.24, *SD* = 0.67), followed by Critical Thinking (*M* = 3.94, *SD* = 0.62), Decision-Making and Problem-Solving (*M* = 3.87, *SD* = 0.63), Self-Awareness (*M* = 3.86, *SD* = 0.75), and Social Responsibility (*M* = 3.84, *SD* = 0.79). Creative Thinking, Interpersonal Relationships and Communication, and Self-Esteem also presented mean scores above the scale midpoint.

Among the positively worded dimensions, Coping with Stress and Emotions showed the lowest mean score (*M* = 3.26, *SD* = 0.76), suggesting comparatively greater perceived difficulties in emotional regulation and stress management. Teamwork presented the lowest overall mean score (*M* = 2.42, *SD* = 0.97); however, because this dimension is negatively worded, lower scores indicate more favourable attitudes towards teamwork. Standard deviations ranged from 0.62 to 0.97, with greater variability observed for Self-Esteem and Teamwork.

### 3.6. Correlations Between LSS Dimensions and Convergent Validity Evidence

Spearman’s rank-order correlations between the LSS dimensions are presented in Table 4. Given the large sample size, interpretation focused on the direction and magnitude of the associations, in addition to statistical significance. Overall, most correlations among the positively scored LSS dimensions were positive and statistically significant, ranging from weak to strong. This pattern indicates that adolescents reporting higher perceived competence in one life skills domain also tended to report higher competence in other related domains.

The strongest association was observed between Self-Awareness and Self-Esteem (*r*_s_ = 0.754, *p* < 0.001), suggesting a close relationship between awareness of one’s own characteristics, abilities, and emotions and perceived self-worth. Strong positive correlations were also found between Critical Thinking and Decision-Making and Problem-Solving (*r*_s_ = 0.670, *p* < 0.001), Interpersonal Relationships and Communication and Self-Awareness (*r*_s_ = 0.659, *p* < 0.001), Creative Thinking and Decision-Making and Problem-Solving (*r*_s_ = 0.624, *p* < 0.001), and Interpersonal Relationships and Communication and Decision-Making and Problem-Solving (*r*_s_ = 0.616, *p* < 0.001). These findings are consistent with the conceptual proximity between cognitive, interpersonal, and self-reflective competencies.

Moderate correlations were observed between Empathy and Social Responsibility (*r*_s_ = 0.577, *p* < 0.001), Creative Thinking and Interpersonal Relationships and Communication (*r*_s_ = 0.576, *p* < 0.001), Self-Awareness and Coping with Stress and Emotions (*r*_s_ = 0.545, *p* < 0.001), Critical Thinking and Creative Thinking (*r*_s_ = 0.535, *p* < 0.001), Interpersonal Relationships and Communication and Empathy (*r*_s_ = 0.533, *p* < 0.001), and Creative Thinking and Self-Awareness (*r*_s_ = 0.529, *p* < 0.001). These associations support the interrelated nature of the cognitive, emotional, interpersonal, and prosocial competencies assessed by the LSS.

The Teamwork dimension showed a distinct pattern, which should be interpreted in light of its negatively worded items. Higher Teamwork scores indicate less favourable attitudes towards teamwork. Accordingly, negative correlations were observed between Teamwork and several positively scored dimensions, including Empathy (*r*_s_ = −0.367, *p* < 0.001), Critical Thinking (*r*_s_ = −0.144, *p* < 0.001), Interpersonal Relationships and Communication (*r*_s_ = −0.132, *p* < 0.001), Decision-Making and Problem-Solving (*r*_s_ = −0.122, *p* < 0.001), and Social Responsibility (*r*_s_ = −0.124, *p* < 0.001). These associations suggest that less favourable attitudes towards teamwork were generally associated with lower perceived competence in related social and cognitive domains.

Some correlations involving Teamwork were negligible or non-significant, namely those with Creative Thinking (*r*_s_ = −0.021, *p* = 0.368) and Self-Esteem (*r*_s_ = 0.028, *p* = 0.240). The association with Self-Awareness was very weak, although statistically significant (*r*_s_ = −0.047, *p* = 0.048), and the association with Coping with Stress and Emotions was also very weak (*r*_s_ = 0.103, *p* < 0.001). These small coefficients should be interpreted cautiously.

Overall, the correlation pattern suggests a coherent network of associations among most LSS dimensions, particularly among self-awareness, self-esteem, decision-making, critical thinking, interpersonal relationships, empathy, and social responsibility. These findings are consistent with the multidimensional but interrelated nature of the life skills assessed by the LSS. Convergent validity evidence was supported by moderate-to-strong positive correlations among LSS dimensions, indicating that they assess related but distinct psychosocial competencies.

To further strengthen the construct validity of the Portuguese version of the LSS, convergent and discriminant validity were also evaluated at the measurement model level. Convergent validity was assessed using the AVE, whereas discriminant validity was examined using the HTMT. AVE values ranged from 0.434 to 0.715. Five factors (Factors 2, 5, 6, 8, and 9) exceeded the recommended threshold of 0.50, while the remaining factors presented values slightly below this criterion. Nevertheless, considering the satisfactory reliability coefficients obtained for all factors (see Internal Consistency section), these findings support partial evidence of convergent validity. Furthermore, HTMT values ranged from 0.063 to 0.842, all below the conservative threshold of 0.85, providing additional support for the discriminant validity of the ten dimensions (Table 5).

### 3.7. Validity Evidence Based on Relations with Depressive and Anxiety Symptoms

Divergent validity evidence was examined through Spearman’s correlations between LSS dimensions and mental health indicators, namely depressive symptoms assessed with the PHQ-9 and anxiety symptoms assessed with the GAD-7 (Table 6). Given the large sample size, the interpretation focused on the direction and magnitude of the associations, as well as statistical significance.

Overall, the pattern of associations provided partial support for the expected relations between life skills and psychological symptomatology. Several LSS dimensions showed negative correlations of low to moderate magnitude with depressive and anxiety symptoms. The strongest negative associations were observed for Coping with Stress and Emotions, which was negatively correlated with depressive symptoms (*r*_s_ = −0.436, *p* < 0.01) and anxiety symptoms (*r*_s_ = −0.473, *p* < 0.01). Self-Esteem also showed negative associations with depressive symptoms (*r*_s_ = −0.461, *p* < 0.01) and anxiety symptoms (*r*_s_ = −0.381, *p* < 0.01), while Self-Awareness was negatively associated with depressive symptoms (*r*_s_ = −0.345, *p* < 0.01) and anxiety symptoms (*r*_s_ = −0.268, *p* < 0.01).

Interpersonal Relationships and Communication showed weaker but statistically significant negative correlations with depressive symptoms (*r*_s_ = −0.221, *p* < 0.01) and anxiety symptoms (*r*_s_ = −0.140, *p* < 0.01). Creative Thinking was weakly and negatively associated with depressive symptoms (*r*_s_ = −0.084, *p* < 0.01), but was not significantly associated with anxiety symptoms. Decision-Making and Problem-Solving showed a very weak negative correlation with depressive symptoms (*r*_s_ = −0.061, *p* < 0.05), but was not significantly associated with anxiety symptoms. Critical Thinking and Teamwork showed no statistically significant associations with either depressive or anxiety symptoms.

Some dimensions showed small positive associations with mental health indicators. Empathy was weakly and positively associated with anxiety symptoms (*r*_s_ = 0.123, *p* < 0.01), but was not significantly associated with depressive symptoms. Social Responsibility was weakly and positively associated with depressive symptoms (*r*_s_ = 0.071, *p* < 0.01) and anxiety symptoms (*r*_s_ = 0.183, *p* < 0.01). Given their small magnitude, these associations should be interpreted cautiously. They may reflect the complexity of prosocial sensitivity, concern for others, and perceived responsibility during adolescence, rather than indicating poorer functioning in these domains.

Taken together, these findings indicate that the LSS dimensions most directly related to emotional regulation and self-evaluation, particularly Coping with Stress and Emotions, Self-Esteem, and Self-Awareness, were more clearly associated with lower depressive and anxiety symptoms. In contrast, several other dimensions showed weak, non-significant, or small positive associations with mental health indicators. This pattern suggests that the LSS assesses psychosocial competencies that are related to, but not redundant with, depressive and anxiety symptoms. Divergent validity evidence was therefore supported by low-to-moderate negative correlations with depressive and anxiety symptoms, indicating that the LSS dimensions are associated with adolescent mental health while remaining conceptually distinct from psychological symptomatology.

### 3.8. Confirmatory Factor Analysis Results

#### 3.8.1. Ten-Factor CFA Model

CFA was conducted in the second randomly selected subsample (*n* = 904) using the MLR estimator. Two models were tested: the original theoretical ten-factor model, comprising 83 items, and the eight-factor model derived from the EFA, comprising 78 items. The path diagrams for the ten-factor and eight-factor CFA models are provided in Supplementary Figures S1 and S2, respectively. Comparative fit indices for both models are presented in Table 7.

For the original ten-factor model, all standardised factor loadings were statistically significant (*p* < 0.001), ranging from λ = 0.444 to λ = 0.849. The highest loading ranges were observed for Self-Awareness (λ = 0.641–0.849), Self-Esteem (λ = 0.548–0.849), Teamwork (λ = 0.481–0.838), and Empathy (λ = 0.655–0.795). Lower loading ranges were observed for Critical Thinking (λ = 0.444–0.709), Social Responsibility (λ = 0.464–0.775), and Coping with Stress and Emotions (λ = 0.477–0.746), although all values remained above 0.40.

The global fit of the ten-factor model was mixed. The chi-square test was statistically significant, χ^2^(3275) = 9243.74, *p* < 0.001. Incremental fit indices were below preferred cut-off values, with CFI = 0.855, TLI = 0.849, NNFI = 0.849, IFI = 0.855, and RNI = 0.855. However, error-based indices indicated acceptable approximate and residual fit, with RMSEA = 0.045, 90% CI [0.044, 0.046], *p*(RMSEA ≤ 0.05) = 1.000, and SRMR = 0.062. The PNFI was 0.762, suggesting adequate parsimony, whereas the GFI was 0.769 and the ECVI was 10.93.

Taken together, these results provide partial support for the original ten-factor structure. Evidence was stronger for item-level performance, RMSEA, and SRMR than for the incremental fit indices. Therefore, the ten-factor model was considered theoretically defensible and empirically preferable to the alternative eight-factor model, while recognising that global model fit was not optimal across all indices.

The latent factor variances in the ten-factor CFA model were all statistically significant (*p* < 0.001), ranging from σ^2^ = 0.184 to σ^2^ = 0.808. The highest variance was observed for Self-Esteem (σ^2^ = 0.808, 95% CI [0.707, 0.910]), followed by Teamwork (σ^2^ = 0.503, 95% CI [0.395, 0.611]) and Self-Awareness (σ^2^ = 0.457, 95% CI [0.367, 0.548]). The lowest variances were observed for Critical Thinking (σ^2^ = 0.184, 95% CI [0.115, 0.252]) and Creative Thinking (σ^2^ = 0.199, 95% CI [0.137, 0.261]). These findings indicate statistically significant variability across all ten latent dimensions assessed by the LSS.

#### 3.8.2. Comparison with the Eight-Factor CFA Model

The alternative eight-factor model derived from the EFA also showed statistically significant standardised factor loadings (*p* < 0.001), ranging from λ = 0.372 to λ = 0.838. The highest loading ranges were found for the combined Self-Esteem/Self-Awareness factor (λ = 0.553–0.835), Teamwork (λ = 0.486–0.838), Interpersonal Relationships and Communication (λ = 0.612–0.790), and the Self-Awareness factor (λ = 0.644–0.775). Lower loadings were observed for some indicators of Social Responsibility and Empathy, particularly items 80 (λ = 0.372) and 82 (λ = 0.461), as well as for selected indicators from Coping with Stress and Emotions and from the Decision-Making, Problem-Solving, and Critical Thinking factor.

The eight-factor model showed less favourable fit than the original ten-factor model. The chi-square test was statistically significant, χ^2^(2897) = 9926.89, *p* < 0.001. Incremental fit indices were below recommended values, with CFI = 0.818, TLI = 0.811, NNFI = 0.811, IFI = 0.819, and RNI = 0.818. The RMSEA was 0.052, 90% CI [0.051, 0.054], and the SRMR was 0.066, both within acceptable ranges. However, the RMSEA test of close fit was statistically significant (*p* < 0.001), indicating that the close-fit assumption was not supported for this model. The GFI was 0.738 and the ECVI was 11.65 (Table 7).

Additional exploratory diagnostics for the eight-factor model also suggested mixed evidence. Some AVE estimates met or approached the recommended 0.50 threshold, whereas others fell below this criterion, indicating heterogeneous evidence of convergent validity across the EFA-derived factors. Modification indices further suggested potential cross-loadings involving items 63, 48, 47, 40, and 60, indicating conceptual overlap among some domains. These findings were interpreted as supplementary evidence and were not used as the main criterion for model selection.

Overall, comparison of the two CFA models indicated that the original ten-factor model provided a more adequate representation of the data than the eight-factor model derived from the EFA. The ten-factor model showed higher CFI, TLI, NNFI, IFI, and RNI values, lower RMSEA and SRMR values, and a lower ECVI. These findings support retaining the original multidimensional structure as the primary scoring framework for the European Portuguese version of the LSS, while acknowledging that the EFA revealed partial overlap and reorganisation among some domains.

### 3.9. Measurement Invariance Across Sex

Measurement invariance across sex was examined among female and male adolescents. The configural model showed good overall fit, χ^2^(6550) = 19,178.945, CFI = 0.979, TLI = 0.979, RMSEA = 0.067, 90% CI [0.066, 0.068], and SRMR = 0.070 (Table 8). Imposing equality constraints on factor loadings resulted in only a small decrease in model fit, with CFI decreasing by 0.003, RMSEA increasing by 0.004, and SRMR increasing by 0.003. These changes supported metric invariance across female and male adolescents. The scalar model showed virtually no additional deterioration in fit compared with the metric model, with no change in CFI or SRMR and a very small decrease in RMSEA (ΔRMSEA = −0.0001) (Table 8), providing preliminary support for scalar invariance across sex. Overall, these findings indicate that the factor structure, factor loadings, and item intercepts of the European Portuguese version of the LSS were broadly comparable across female and male adolescents, providing preliminary support for the appropriateness of between-group comparisons in this sample.

### 3.10. Reliability Analysis

Internal consistency estimates for the total LSS and for the ten dimensions retained as the primary scoring framework are presented in Table 9. The total LSS showed excellent internal consistency (Cronbach’s α = 0.963; McDonald’s ω = 0.964). Given the instrument’s length and multidimensional nature, this coefficient was interpreted as evidence of overall internal consistency rather than of unidimensionality.

Across the ten dimensions, Cronbach’s α ranged from 0.782 to 0.936, and McDonald’s ω ranged from 0.783 to 0.936. The highest reliability estimates were observed for Self-Awareness (α = 0.936; ω = 0.936), Self-Esteem (α = 0.929; ω = 0.931), and Decision-Making and Problem-Solving (α = 0.908; ω = 0.907). The lowest, although still satisfactory, estimates were observed for Social Responsibility (α = 0.782; ω = 0.783) and Critical Thinking (α = 0.785; ω = 0.785). Overall, the results supported satisfactory to excellent internal consistency for the European Portuguese version of the LSS.

### 3.11. Final Structure and Scoring Framework of the European Portuguese LSS

Based on the combined exploratory, confirmatory, and reliability evidence, the European Portuguese version of the LSS was retained as an 83-item instrument organised according to the original ten theoretical dimensions: Critical Thinking, Creative Thinking, Decision-Making and Problem-Solving, Coping with Stress and Emotions, Interpersonal Relationships and Communication, Empathy, Self-Awareness, Self-Esteem, Teamwork, and Social Responsibility.

This decision was supported by the theoretical coherence of the original LSS structure, the statistically significant CFA factor loadings across all items, the comparatively better fit of the ten-factor model relative to the eight-factor model, and the satisfactory-to-excellent internal consistency estimates observed for the total scale and its dimensions. Although the EFA suggested an interpretable eight-factor solution, the CFA did not support this structure as a superior alternative to the original model.

Nevertheless, the partial reorganisation observed in the EFA, the lower-than-ideal incremental fit indices in the ten-factor CFA model, and the items with weaker loadings, higher uniqueness values, or possible cross-loading indicate that the dimensional structure of the European Portuguese LSS should be further examined in future Portuguese samples. At this stage, no items were removed because all items were theoretically relevant, contributed to the content coverage of the original LSS, and showed statistically significant loadings in the retained ten-factor CFA model. Future studies should reassess these items before proposing a shortened or revised version of the scale.

## 4. Discussion

### 4.1. Principal Findings

This study translated, culturally adapted, and examined the psychometric properties of the European Portuguese version of the LSS in a large school-based sample of Portuguese adolescents. Overall, the findings provide initial support for the European Portuguese LSS as a multidimensional instrument for assessing adolescents’ cognitive, emotional, interpersonal, self-evaluative, and prosocial life skills. The adaptation process followed international recommendations and included forward translation, back-translation, review by the original authors, expert panel assessment, and pilot testing with adolescents, supporting the semantic, conceptual, and cultural adequacy of the adapted version.

The EFA indicated excellent suitability for factor extraction and suggested an interpretable eight-factor solution explaining 50.7% of the total variance. This exploratory structure was broadly coherent with the conceptual framework of the original LSS, although some dimensions showed partial overlap and reorganisation. This was particularly evident for Critical Thinking with Decision-Making and Problem-Solving, Self-Esteem with Self-Awareness, and Social Responsibility with Empathy. These findings may reflect the close conceptual and developmental relationships among life skills during adolescence, as these competencies are expected to operate in an integrated and mutually supportive manner. However, the exploratory findings do not, by themselves, establish that the underlying construct is organised differently in Portuguese adolescents.

CFA was then used to compare the original ten-factor model with the EFA-derived eight-factor model. Although neither model showed optimal fit across all indices, the ten-factor model provided a comparatively better representation of the data, with statistically significant standardised loadings across all items and more favourable fit indices than the eight-factor model. RMSEA and SRMR supported acceptable approximate and residual fit, whereas CFI and TLI remained below preferred thresholds. Therefore, the original ten-dimensional structure was retained as the primary scoring framework at this stage, as it provided the best balance between theoretical coherence, empirical evidence, and cross-cultural comparability, while still requiring further examination in independent samples. Reliability analyses further supported the internal consistency of the European Portuguese LSS. The total scale showed excellent reliability, and all ten dimensions presented satisfactory to excellent Cronbach’s alpha and McDonald’s omega values. Additional evidence of construct validity was obtained through AVE and HTMT analyses. Although AVE values for some factors were slightly below the recommended threshold of 0.50, they were accompanied by satisfactory reliability coefficients, providing partial evidence of convergent validity. All HTMT values were below the conservative threshold of 0.85, providing further support for the discriminant validity of the ten dimensions. The supplementary measurement invariance analysis also provided preliminary support for configural, metric, and scalar invariance across female and male adolescents, providing preliminary support for the appropriateness of between-group comparisons in the present sample.

Validity evidence based on relations with other variables was partially supported by the pattern of associations with depressive and anxiety symptoms. Dimensions more directly related to emotional regulation and self-evaluation, particularly Coping with Stress and Emotions, Self-Esteem, and Self-Awareness, showed the strongest negative associations with PHQ-9 and GAD-7 scores. In contrast, cognitive, interpersonal, and prosocial dimensions showed weaker or negligible direct associations with depression and anxiety indicators. This pattern is theoretically coherent, as these competencies may contribute to adolescent functioning through broader educational, interpersonal, and developmental pathways rather than through direct associations with current internalising symptoms. Taken together, these findings support retaining the original ten-dimensional structure as the primary scoring framework at this stage, while recognising the need for further examination of item functioning, factorial stability, and measurement equivalence in independent and more diverse adolescent samples.

### 4.2. Factor Structure and Comparison with the Original LSS

The original LSS was developed as an 83-item instrument comprising ten subdimensions: Critical Thinking, Creative Thinking, Decision-Making and Problem-Solving, Coping with Stress and Emotions, Interpersonal Relationships and Communication, Empathy, Self-Awareness, Self-Esteem, Teamwork, and Social Responsibility (Erduran Avcı & Korur, 2022). In the present study, the EFA suggested partial reorganisation of this structure. Items from Decision-Making and Problem-Solving and Critical Thinking clustered together, suggesting that Portuguese adolescents may interpret reflective reasoning, evaluation of alternatives, and decision-making as closely related cognitive competencies. Similarly, Social Responsibility and Empathy items loaded together, indicating conceptual proximity between concern for others, prosocial sensitivity, and responsibility for the consequences of one’s actions. Some overlap between Self-Esteem and Self-Awareness was also observed, which is theoretically plausible during adolescence.

The partial reorganisation observed in the EFA may therefore reflect the interrelated nature of psychosocial competencies during adolescence. Critical Thinking and Decision-Making/Problem-Solving may be closely connected because adolescents often use reflective reasoning, evaluation of alternatives, and problem-solving jointly when responding to everyday challenges. Recent conceptual work similarly identifies decision-making and problem-solving as central attributes of adolescent life skills within school and social contexts (Torres et al., 2025). The overlap between Self-Awareness and Self-Esteem may also be developmentally plausible, as adolescence involves ongoing identity exploration and the consolidation of self-concept clarity and self-evaluation (Avci et al., 2025). Finally, the proximity between Empathy and Social Responsibility may reflect their shared involvement in prosocial functioning, as meta-analytic evidence demonstrates a positive association between empathy and prosocial behaviour during adolescence (Li et al., 2025). These patterns may reflect developmental characteristics of adolescence, cultural influences on how psychosocial competencies are understood and expressed, and/or possible limitations of the original theoretical taxonomy in clearly distinguishing between closely related life skill domains.

Despite the interpretability of the eight-factor solution, CFA results favoured the original ten-factor model. The ten-factor model showed statistically significant standardised factor loadings across all items and a comparatively better fit than the EFA-derived eight-factor model. RMSEA and SRMR supported acceptable approximate and residual fit, although CFI and TLI, and related incremental fit indices, remained below conventional thresholds for good fit. The ten-factor model also showed a lower ECVI than the eight-factor model, supporting its comparatively better performance. Conversely, the eight-factor model showed weaker incremental fit indices and a higher ECVI. Supplementary diagnostics for the eight-factor model also suggested heterogeneous convergent validity and possible cross-loadings, indicating that some EFA-derived factors may require further refinement.

Therefore, the CFA should be interpreted as providing partial, rather than definitive, support for the original structure. The original ten-factor model appears theoretically defensible and empirically preferable to the alternative eight-factor solution, but the available evidence does not allow its factorial structure to be considered fully confirmed in Portuguese adolescents. The partial convergence observed between some dimensions should not be interpreted as evidence that these competencies are indistinguishable. Rather, it may reflect the functional interdependence of life skills, which are conceptually distinct but developmentally and behaviourally interconnected during adolescence.

It is important to note that neither the ten-factor nor the eight-factor model achieved optimal fit across all indices. Although the ten-factor model was comparatively preferable and theoretically coherent, the lower-than-desired CFI and TLI values suggest that the structure of the European Portuguese LSS may not be fully captured by a standard correlated-factor CFA model. This may reflect the complexity of the construct, conceptual overlap among life skill domains, potential cross-loadings, correlated residuals, or the presence of higher-order or bifactor structures that were not tested in the present study. Therefore, the retained ten-dimensional scoring framework should be interpreted as an initial, theoretically grounded solution that requires further examination in independent Portuguese adolescent samples.

Future research should continue evaluating the factorial structure of the European Portuguese LSS using more flexible structural modelling approaches. Exploratory structural equation modelling (ESEM) may better accommodate cross-loadings among conceptually related domains, whereas bifactor models could clarify the extent to which adolescents’ responses are explained by a general life-skills factor in addition to specific dimensions. Future studies should also examine item refinement, factorial stability, and measurement invariance beyond female and male adolescents, including age groups, gender identity, nationality, migration background, socioeconomic background, and different educational contexts.

### 4.3. Interpretation of Item-Level Findings

At the item level, most LSS items showed adequate associations with their respective factors, supporting their contribution to assessing adolescents’ psychosocial competencies. However, some items presented higher uniqueness values or weaker factor loadings, suggesting that they were less strongly represented by the extracted factor structure. These included items related to changing one’s thinking when presented with evidence, projecting negative emotions onto others, critically evaluating one’s actions, setting aside perfectionism, volunteering for socially useful activities, and trying different ways of coping with stress.

These findings should be interpreted cautiously. Higher uniqueness values do not necessarily imply that an item should be removed, particularly in a broad multidimensional instrument designed to cover several life skill domains. Some items may capture context-dependent behaviours, socially desirable responses, or competencies that adolescents interpret in heterogeneous ways. Items from Interpersonal Relationships and Communication and Empathy that showed weaker loadings may also reflect conceptual proximity between communication, empathy, social responsibility, and self-awareness during adolescence. These items were retained because of their theoretical relevance and contribution to content coverage, but future studies should examine them using complementary approaches, such as cognitive interviewing, item response theory, differential item functioning, and measurement invariance analyses.

### 4.4. Reliability of the European Portuguese LSS

The European Portuguese version of the LSS demonstrated satisfactory to excellent internal consistency. The total scale showed Cronbach’s α = 0.963 and McDonald’s ω = 0.964. Given that the LSS is a multidimensional instrument comprising ten distinct domains, these overall coefficients should be interpreted as indicators of the instrument’s overall reliability rather than evidence of unidimensionality. Reliability estimates for the individual dimensions ranged from satisfactory to excellent, with all coefficients above 0.78, supporting the internal consistency of each subscale.

The highest reliability coefficients were observed for the Self-Awareness, Self-Esteem, and Decision-Making and Problem-Solving dimensions, whereas Social Responsibility and Critical Thinking showed the lowest, albeit still satisfactory, values. The Teamwork dimension also demonstrated satisfactory reliability despite consisting exclusively of negatively worded items. Overall, these findings are consistent with those reported in the original validation study and support the reliability of the ten-dimensional scoring framework retained for the European Portuguese version of the LSS.

Although the high Cronbach’s alpha and McDonald’s omega coefficients provide strong evidence of internal consistency, values exceeding 0.90 may also suggest a degree of item redundancy (Tavakol & Dennick, 2011). Because the primary aim of this study was to translate, culturally adapt, and examine the psychometric properties of the original instrument while preserving its theoretical and factorial structure, no item reduction procedures were undertaken. Consequently, the potential redundancy of some items should be acknowledged as a limitation of the present study. Future research should investigate whether a more parsimonious version of the scale can be achieved through systematic item reduction while preserving the conceptual representation of the ten-life skill domains. Any shortened version should subsequently be evaluated through confirmatory factor analysis in an independent sample to ensure that its factorial structure and psychometric properties remain robust.

### 4.5. Associations Between Life Skills and Mental Health Indicators

Validity evidence based on relations with other variables was examined through correlations between LSS dimensions and symptoms of depression and anxiety. The strongest and most theoretically consistent negative associations were observed for Coping with Stress and Emotions, Self-Esteem, and Self-Awareness. Adolescents reporting higher levels in these dimensions tended to report fewer depressive and anxiety symptoms. This pattern is consistent with the conceptualisation of life skills as psychosocial competencies that support emotional regulation, self-understanding, adaptive coping, and psychological well-being. Meta-analytic evidence further indicates that self-esteem/self-concept is strongly negatively associated with depressive symptoms in youth, while self-esteem, self-awareness, self-efficacy, and self-regulation show moderate negative associations with anxiety (Yeo et al., 2023).

Coping with Stress and Emotions showed the strongest association with anxiety symptoms and was also moderately associated with depressive symptoms. This dimension directly reflects adolescents’ perceived ability to manage stress, negative emotions, anger, anxiety, and emotionally challenging situations, which may explain its comparatively closer relationship with internalising symptoms. This interpretation is consistent with systematic-review evidence showing that adolescents’ beliefs about their capacity to regulate or control emotions are associated with fewer symptoms of anxiety and depression, partly through greater use of adaptive emotion-regulation strategies (Somerville et al., 2024). Self-Esteem also showed meaningful negative associations with both depressive and anxiety symptoms, while Self-Awareness was similarly associated with lower symptom levels. Together, these findings suggest that competencies involving emotional regulation and self-evaluation may be particularly relevant to adolescents’ psychological adjustment. However, because the present study was cross-sectional, these associations should not be interpreted as evidence that having more life skills reduces depressive or anxiety symptoms, or vice versa.

Interpersonal Relationships and Communication showed weaker but statistically significant negative associations with both depression and anxiety. Although smaller in magnitude, these findings are consistent with the potential relevance of interpersonal competence and effective communication to adolescents’ social functioning and well-being. The comparatively weaker associations may also indicate that interpersonal skills contribute to adolescent adjustment through broader relational and social pathways that are not fully captured by concurrent measures of internalising symptoms.

In contrast, Critical Thinking and Teamwork were not significantly associated with depressive or anxiety symptoms, while Creative Thinking and Decision-Making and Problem-Solving showed only very weak or non-significant associations. These findings suggest that not all life skills are expected to show direct associations with current psychological symptomatology. Cognitive and behavioural competencies may instead contribute to adolescent functioning through educational, interpersonal, decision-making, or longer-term developmental pathways. This pattern therefore supports the conceptual distinction between life skills and mental health symptoms: the LSS assesses a broader set of psychosocial competencies rather than functioning as an indirect measure of depression or anxiety.

Of particular interest, Empathy showed a small positive association with anxiety symptoms, despite showing no significant association with depressive symptoms. Although this finding may initially appear counterintuitive, it may be compatible with the multidimensional nature of empathy. Empathic responding involves cognitive and affective processes, and conceptualisations of empathy also recognise personal distress as an aversive emotional response that may arise when experiencing or witnessing others’ emotional states (Li et al., 2025). Accordingly, greater sensitivity to others’ emotions may, in some adolescents, coexist with heightened emotional distress rather than uniformly reflecting better psychological adjustment. At the same time, meta-analytic evidence indicates that empathy is positively associated with prosocial behaviour during adolescence, reinforcing the possibility that empathy may be beneficial for social functioning while having more complex relationships with internalising experiences (Li et al., 2025).

This interpretation should nevertheless remain tentative. The Empathy dimension of the LSS does not distinguish cognitive empathy, affective empathy, and personal distress, and the present findings therefore cannot determine which component may account for the observed positive association with anxiety. Future studies combining the LSS with more differentiated measures of empathy could help clarify whether specific forms of empathic responding are differently associated with adolescents’ psychological adjustment. Li et al. describe cognitive, affective, and personal-distress components as conceptually distinguishable but interrelated aspects of empathic responding (Li et al., 2025), providing a useful framework for examining this possibility.

Social Responsibility also showed small positive associations with depressive and anxiety symptoms. Given their weak magnitude, these findings should be interpreted cautiously and should not be taken to indicate that social responsibility is detrimental to adolescent mental health. One possible interpretation is that heightened concern for others, perceived obligations towards others, or sensitivity to social consequences may coexist with greater emotional burden in some adolescents. However, unlike the interpretation proposed for empathy, the present study does not provide evidence regarding the mechanisms underlying these associations. Further research is therefore needed to determine whether these findings are replicated and whether they reflect developmental, contextual, measurement-related, or other psychosocial processes.

Overall, the pattern of associations provides partial support for validity evidence based on relations with mental health indicators. Dimensions most directly related to emotional regulation and self-evaluation, particularly Coping with Stress and Emotions, Self-Esteem, and Self-Awareness showed the clearest negative associations with depressive and anxiety symptoms, whereas cognitive, interpersonal, and prosocial competencies demonstrated weaker, null, or, in a small number of cases, positive associations. This differentiated pattern supports the interpretation of the LSS as a multidimensional measure of psychosocial competencies that are related to, but conceptually distinct from, adolescent psychological symptomatology.

### 4.6. Implications for School-Based Practice and Adolescent Mental Health Promotion

The findings have relevant implications for school-based practice, health promotion, and adolescent mental health support. Schools are central contexts for adolescent development and provide accessible settings for universal and selective interventions to strengthen psychosocial competencies. A culturally adapted measure such as the European Portuguese LSS may help teachers, school nurses, psychologists, youth workers, and other professionals identify adolescents’ strengths and difficulties across multiple life skill domains.

The LSS may be useful for planning and monitoring school-based interventions focused on emotional regulation, decision-making, communication, empathy, self-awareness, and responsible social behaviour. Because the scale covers cognitive, emotional, and social competencies, it can support a broader understanding of adolescent well-being beyond symptom reduction alone. The associations with depression and anxiety further suggest that Coping with Stress and Emotions, Self-Esteem, Self-Awareness, and Interpersonal Relationships and Communication may be particularly relevant domains for interventions aiming to reduce vulnerability to psychological distress.

For professionals working with adolescents, the LSS may support assessment, programme planning, needs identification, and evaluation of intervention outcomes. The instrument may also contribute to integrated responses across schools, community programmes, school health services, and youth support services, particularly in initiatives aimed at promoting adolescent well-being, strengthening psychosocial competencies, and identifying areas requiring additional support.

From a policy perspective, these findings may support the integration of life skills assessment into broader adolescent health promotion and school mental health strategies (Johns Hopkins Bloomberg School of Public Health & United Nations Children’s Fund, 2022; Taylor et al., 2017; WHO, 2020, 2025). A culturally adapted measure of life skills may help policymakers, school health teams, and educational stakeholders identify priority areas for intervention, monitor adolescents’ psychosocial competencies, and evaluate the impact of school- and community-based programmes. In this sense, the LSS may contribute to evidence-informed planning of preventive interventions aimed at strengthening emotional regulation, coping, interpersonal communication, self-awareness, and self-esteem among adolescents.

### 4.7. Strengths and Limitations

This study has several strengths. First, it used a large school-based sample of Portuguese adolescents. It randomly divided the sample into independent subsamples for EFA and CFA, thereby reducing the risk of capitalisation on chance and enabling cross-validation of the factorial structure. Second, the translation and cultural adaptation process followed internationally recognised guidelines and included forward and backward translation, expert panel review, pilot testing, and approval of the back-translated version by the original developers of the LSS. Third, the study provided a comprehensive psychometric evaluation by examining evidence based on internal structure through both EFA and CFA, assessing internal consistency using Cronbach’s alpha and McDonald’s omega, examining convergent and discriminant validity through AVE and HTMT, and exploring associations among LSS dimensions and with indicators of depression and anxiety.

Several limitations should also be acknowledged. The convenience sample was recruited from public secondary schools in the Central region of Portugal, which may limit the generalisability of the findings to adolescents from other geographical regions, private schools, vocational settings, or out-of-school populations. Although the Central region includes both urban and rural municipalities and encompasses adolescents from diverse socioeconomic backgrounds, it may not fully reflect the demographic, cultural, educational, and socioeconomic diversity of the Portuguese adolescent population. Regional differences in educational practices, school resources, family socioeconomic conditions, cultural norms, and opportunities for psychosocial development may influence how adolescents interpret and respond to life-skills items. Consequently, the psychometric properties observed in the present study should not be assumed to be directly transferable to all Portuguese adolescents without further empirical verification.

Although the sample was predominantly Portuguese, it included adolescents from different national backgrounds. Subgroup analyses by nationality were not conducted because the non-Portuguese groups were comparatively small and unevenly distributed, which would limit the robustness and interpretability of between-group comparisons. Therefore, the findings should be interpreted with caution regarding their applicability to adolescents from different nationalities or migration backgrounds. Future studies should replicate the validation process in samples drawn from different Portuguese regions and include larger, more balanced multicultural samples. These studies should examine measurement invariance and potential differences in LSS scores across nationality, migration background, cultural groups, urban and rural settings, public and private schools, vocational education contexts, and socioeconomically diverse populations, to strengthen the external validity and national applicability of the European Portuguese version of the LSS.

Because recruitment and data collection took place over two academic years, potential cohort-related differences between participants assessed at different times cannot be entirely ruled out. The cross-sectional design prevents causal conclusions regarding associations between life skills and symptoms of depression and anxiety. In addition, all measures were self-reported and collected within the same assessment context, which may have increased the risk of common method variance and social desirability bias.

Another limitation concerns the factorial structure. Although the original ten-factor model demonstrated better performance than the alternative eight-factor solution derived from the EFA, the confirmatory findings provided partial rather than definitive support for the original theoretical structure. Specifically, while the RMSEA and SRMR indicated acceptable approximate model fit, the incremental fit indices (CFI, TLI, IFI, NNFI, and RNI) remained below the values generally recommended for well-fitting measurement models. These findings suggest that the retained ten-dimensional structure should be regarded as theoretically defensible and psychometrically acceptable, but not yet fully confirmed within the Portuguese adolescent population. Consequently, further validation studies using independent samples are warranted before stronger conclusions regarding the factorial structure can be drawn.

The complexity of the life skills construct, and the conceptual overlap among several domains may not be fully captured by a conventional correlated-factor CFA model. Furthermore, some items exhibited relatively high uniqueness values, comparatively weaker factor loadings, or indications of cross-loadings. Although Varimax rotation was retained to facilitate comparison with the original validation study and support interpretability, the observed interrelations among LSS dimensions suggest that alternative exploratory approaches based on oblique rotations may provide additional insights into the instrument’s underlying structure. Accordingly, future research should compare orthogonal and oblique exploratory solutions and evaluate more flexible structural modelling approaches, particularly ESEM and bifactor models, to determine whether these provide a more accurate representation of the latent structure of the European Portuguese LSS.

In addition, the EFA and CFA treated five-point Likert-type item responses as approximately continuous. Although this approach is commonly adopted in psychometric research, future studies should examine whether the present findings are replicated using polychoric correlation matrices and ordinal estimation methods, such as weighted least squares mean and variance-adjusted (WLSMV) or diagonally weighted least squares (DWLS). No sensitivity analysis excluding the items that did not reach the predefined loading criterion in the EFA was conducted; therefore, future research should examine whether removing or revising these items improves model fit without compromising the theoretical and content coverage of the LSS. Future studies should also investigate the refinement of items with comparatively weaker psychometric performance, confirm the factorial structure in independent Portuguese samples, and assess measurement invariance across relevant demographic groups, including gender identity, age, nationality, migration background, socioeconomic background, and educational contexts, to determine whether the instrument operates equivalently across adolescent subpopulations. In addition, future research should examine differential item functioning (DIF) to determine whether individual items perform equivalently across demographic groups, including sex/gender, age, nationality, migration background, and educational contexts.

The length of the 83-item LSS may also represent a practical limitation, particularly in educational and clinical settings where assessment time and adolescents’ attentional engagement may be limited. Although the average completion time was acceptable, administering a relatively long questionnaire may increase the risk of response fatigue and reduced attentional engagement. In addition, reliability was examined only through internal consistency estimates; therefore, future studies should also assess test–retest reliability and longitudinal stability to provide further evidence of the instrument’s temporal stability. Moreover, the very high Cronbach’s alpha and McDonald’s omega values observed for the total scale and some dimensions may indicate a degree of item redundancy. Future research should therefore examine whether a more parsimonious version of the LSS can be developed through systematic item reduction while preserving the conceptual representation of the ten life skill domains. Any shortened version should subsequently be evaluated through confirmatory factor analysis in an independent sample to ensure that its factorial structure and psychometric properties remain robust.

Finally, although the PHQ-9 and GAD-7 provided useful external criteria for examining construct validity, the present study did not aim to provide a detailed clinical characterisation of depressive and anxiety symptom severity in the sample. Future validation studies would benefit from including a broader range of external constructs and clinical samples to further strengthen the evidence for the construct validity and generalisability of the European Portuguese version of the LSS.

### 4.8. Future Research

Future research should initially focus on further validating the European Portuguese LSS within Portugal. Independent studies should replicate the factorial structure in adolescent samples recruited from different regions of mainland Portugal and the Autonomous Regions of the Azores and Madeira, and should include a broader range of educational settings, including public, private, and vocational schools, as well as urban and rural contexts. Within Portuguese samples, measurement invariance should be further examined across sex, gender identity, age groups, school years, socioeconomic backgrounds, nationality, and migration background. Future psychometric studies should also assess item functioning, differential item functioning, test–retest reliability, longitudinal stability, and the robustness of the factorial structure using ordinal estimation methods.

Further research in Portugal should also compare alternative structural models, including oblique exploratory solutions, ESEM, higher-order models, and bifactor models, to determine whether these approaches provide a more accurate representation of the interrelations among LSS dimensions. The development of a shorter version of the scale may also be considered if specific items consistently demonstrate weak loadings, high uniqueness values, cross-loadings, or evidence of redundancy. Any shortened version should be developed and subsequently validated in independent Portuguese adolescent samples.

Once the factorial structure and measurement properties of the European Portuguese LSS have been replicated across diverse Portuguese samples, a subsequent line of research could examine the broader cross-cultural applicability of the original LSS framework in other European adolescent populations. Such studies would require appropriate translation and cultural adaptation of the instrument for each linguistic and cultural context, followed by country-specific psychometric evaluation and cross-national measurement invariance testing before direct comparisons between countries are undertaken.

## 5. Conclusions

This study provides initial psychometric evidence for the European Portuguese version of the LSS in a large school-based sample of adolescents. The translation and cultural adaptation process followed international guidelines and supported the linguistic, semantic, conceptual, and cultural adequacy of the instrument for use in this population.

The findings support the LSS as a promising multidimensional instrument for assessing life skills among Portuguese adolescents. However, the factorial structure should be interpreted cautiously, as the ten-factor model showed better performance than the alternative eight-factor solution but received only partial support across fit indices. Internal consistency was satisfactory to excellent, although very high reliability coefficients may indicate some item redundancy. Additional analyses provided partial evidence of convergent validity and support for discriminant validity, preliminary measurement invariance across female and male adolescents, and theoretically meaningful associations with indicators of depression and anxiety.

The European Portuguese LSS may inform school-based health promotion, psychosocial assessment, adolescent mental health support, and evidence-informed planning by policy-makers and educational and health stakeholders. Future research should prioritise replication of the factorial structure, measurement invariance, temporal stability, item functioning, and potential item reduction in independent and geographically and sociodemographically diverse Portuguese adolescent samples. Subsequent cross-cultural studies may then examine the applicability and measurement equivalence of appropriately adapted versions of the LSS across other European adolescent populations.

## Figures and Tables

**Table 1:** Demographic Characteristics of Participants (*N* = 1807).

Variable	Category	Frequency (*n*)	Percentage (%)
Age	13 years	1	0.1
	14 years	28	1.6
	15 years	478	27
	16 years	562	31.8
	17 years	542	30.6
	18 years	145	8.2
	19 years	13	0.7
Nationality	Portuguese	1640	91.1
	Brazilian	110	6.1
	Other nationalities	51	2.8
Sex assigned at birth	Female	1049	58.3
	Male	713	39.6
	Intersex	11	0.6
	Prefer not to answer	27	1.5
Gender identity	Cisgender	1653	93.3
	Non-binary person	16	0.9
	Trans person	21	1.2
	Prefer not to answer	81	4.6
Year of schooling	10th grade	737	41.6
	11th grade	543	30.7
	12th grade	491	27.7
Self-rated academic performance	Very good	348	19.7
	Good	967	54.7
	Satisfactory	411	23.3
	Unsatisfactory	28	1.6
	Very unsatisfactory	13	0.7
Performance satisfaction	Very satisfied	214	12.1
	Satisfied	910	51.4
	Neither satisfied nor dissatisfied	483	27.3
	Unsatisfied	135	7.6
	Very dissatisfied	27	1.5
Mother’s education	No schooling	7	0.4
	1st cycle	53	3.0
	2nd cycle	189	10.7
	3rd cycle	297	16.8
	Secondary	532	30.1
	Higher education	597	33.7
	Prefer not to answer	95	5.4
Father’s education	No schooling	8	0.5
	1st cycle	79	4.5
	2nd cycle	254	14.4
	3rd cycle	361	20.4
	Secondary	498	28.2
	Higher education	419	23.7
	Prefer not to answer	150	8.5
School social support level	Level A (highest financial support)	117	6.6
	Level B	236	13.4
	Level C	248	14.1
	No assigned level	1163	65.9
Extracurricular activities	Yes	1113	63.1
	No	650	36.9

Note. Percentages are based on valid responses; missing responses varied across variables.

**Table 2:** Initial eigenvalues and explained variance for the eight-factor exploratory solution of the LSS (*n* = 903).

Factor	Eigenvalue	% of Variance	Cumulative %
1	23.630	12.0	12.0
2	6.757	8.7	20.7
3	3.566	6.3	26.9
4	2.284	6.1	33.0
5	1.997	5.8	38.8
6	1.541	5.0	43.8
7	1.227	4.1	47.9
8	1.050	2.8	50.7

Note. LSS = Life Skills Scale. Eigenvalues refer to the initial solution; percentages of variance refer to the rotated factor solution. Factors were retained based on initial eigenvalues greater than 1, inspection of the scree plot, explained variance, and theoretical interpretability.

**Table 3:** Descriptive statistics for the LSS dimensions according to the original ten-dimensional scoring structure.

Dimension	Min	Max	*M*	*SD*
Critical Thinking	1.00	5.00	3.94	0.62
Creative Thinking	1.00	5.00	3.76	0.63
Decision-Making and Problem-Solving	1.00	5.00	3.87	0.63
Coping with Stress and Emotions	1.00	5.00	3.26	0.76
Interpersonal Relationships and Communication	1.00	5.00	3.74	0.68
Empathy	1.00	5.00	4.24	0.67
Self-Awareness	1.00	5.00	3.86	0.75
Self-Esteem	1.00	5.00	3.68	0.89
Teamwork	1.00	5.00	2.42	0.97
Social Responsibility	1.00	5.00	3.84	0.79

Note. Min = minimum; Max = maximum; *M* = mean; *SD* = standard deviation. Scores range from 1 to 5. Higher scores indicate higher perceived life skills, except for Teamwork, whose items are negatively worded; therefore, lower scores indicate more favourable attitudes towards teamwork.

**Table 4:** Spearman correlations between LSS dimensions.

Variable	1	2	3	4	5	6	7	8	9	10
1. Critical Thinking	—									
2. Creative Thinking	0.535 **	—								
3. Decision-Making and Problem-Solving	0.670 **	0.624 **	—							
4. Coping with Stress and Emotions	0.269 **	0.381 **	0.423 **	—						
5. Interpersonal Relationships and Communication	0.481 **	0.576 **	0.616 **	0.535 **	—					
6. Empathy	0.495 **	0.399 **	0.581 **	0.219 **	0.533 **	—				
7. Self-Awareness	0.409 **	0.529 **	0.550 **	0.545 **	0.659 **	0.438 **	—			
8. Self-Esteem	0.296 **	0.489 **	0.435 **	0.589 **	0.585 **	0.280 **	0.754 **	—		
9. Teamwork	−0.144 **	−0.021	−0.122 **	0.103 **	−0.132 **	−0.367 **	−0.047 *	0.028	—	
10. Social Responsibility	0.396 **	0.366 **	0.436 **	0.101 **	0.337 **	0.577 **	0.265 **	0.197 **	−0.124 **	—

Note. Spearman’s rank-order correlation coefficients are reported. * *p* < 0.05; ** *p* < 0.001. Teamwork items are negatively worded; therefore, higher scores indicate less favourable attitudes towards teamwork, which should be considered when interpreting correlations involving this dimension.

**Table 5:** Heterotrait-monotrait ratio.

Variable	1	2	3	4	5	6	7	8	9	10
1. Critical Thinking	—									
2. Creative Thinking	0.710	—								
3. Decision-Making and Problem-Solving	0.842	0.750	—							
4. Coping with Stress and Emotions	0.340	0.462	0.475	—						
5. Interpersonal Relationships and Communication	0.652	0.695	0.742	0.670	—					
6. Empathy	0.666	0.453	0.676	0.265	0.660	—				
7. Self-Awareness	0.491	0.577	0.617	0.577	0.744	0.468	—			
8. Self-Esteem	0.372	0.545	0.494	0.629	0.620	0.247	0.812	—		
9. Teamwork	0.126	0.139	0.092	0.165	0.063	0.313	0.082	0.109	—	
10. Social Responsibility	0.512	0.400	0.508	0.154	0.366	0.635	0.246	0.140	0.149	—

**Table 6:** Spearman correlations between LSS dimensions and depressive and anxiety symptoms.

LSS Dimension	PHQ-9	GAD-7
Critical Thinking	−0.009	0.036
Creative Thinking	−0.084 **	−0.034
Decision-Making and Problem-Solving	−0.061 *	0.012
Coping with Stress and Emotions	−0.436 **	−0.473 **
Interpersonal Relationships and Communication	−0.221 **	−0.140 **
Empathy	0.014	0.123 **
Self-Awareness	−0.345 **	−0.268 **
Self-Esteem	−0.461 **	−0.381 **
Teamwork	0.029	−0.030
Social Responsibility	0.071 **	0.183 **

Note. PHQ-9 = Patient Health Questionnaire-9; GAD-7 = Generalized Anxiety Disorder-7. Higher PHQ-9 and GAD-7 scores indicate higher depressive and anxiety symptoms, respectively. Spearman’s rank-order correlation coefficients are reported. * *p* < 0.05; ** *p* < 0.01. Teamwork items are negatively worded; therefore, higher scores indicate less favourable attitudes towards teamwork, which should be considered when interpreting correlations involving this dimension.

**Table 7:** CFA fit indices for the ten-factor and eight-factor LSS models.

Fit Index	Ten-Factor Model	Eight-Factor Model
χ^2^ (df)	9243.74 (3275)	9926.89 (2897)
p	<0.001	<0.001
CFI	0.855	0.818
TLI	0.849	0.811
NNFI	0.849	0.811
IFI	0.855	0.819
RNI	0.855	0.818
PNFI	0.762	0.735
RMSEA	0.045	0.052
90% CI for RMSEA	[0.044, 0.046]	[0.051, 0.054]
*p*(RMSEA ≤ 0.05)	1.000	<0.001
SRMR	0.062	0.066
GFI	0.769	0.738
ECVI	10.93	11.65

Note. CFI = comparative fit index; TLI = Tucker–Lewis index; NNFI = non-normed fit index; IFI = incremental fit index; RNI = relative noncentrality index; PNFI = parsimony normed fit index; RMSEA = root mean square error of approximation; CI = confidence interval; SRMR = standardised root mean square residual; GFI = goodness-of-fit index; ECVI = expected cross-validation index. Lower ECVI values indicate better comparative fit between competing models.

**Table 8:** Measurement invariance of the European Portuguese version of the LSS across sex.

Model	χ^2^	df	CFI	TLI	RMSEA	90% CI for RMSEA	SRMR	ΔCFI	ΔRMSEA	ΔSRMR
Configural	19,178.945	6550	0.9793	0.9785	0.0669	[0.0658, 0.0680]	0.0701	—	—	—
Metric	21,002.416	6613	0.9764	0.9757	0.0711	[0.0700, 0.0721]	0.0735	−0.0029	+0.0041	+0.0034
Scalar	21,002.416	6623	0.9764	0.9758	0.0710	[0.0699, 0.0721]	0.0735	0.0000	−0.0001	0.0000

Note. LSS = Life Skills Scale; CFI = comparative fit index; TLI = Tucker–Lewis index; RMSEA = root mean square error of approximation; SRMR = standardised root mean square residual; CI = confidence interval. ΔCFI, ΔRMSEA, and ΔSRMR represent changes relative to the preceding, less constrained model.

**Table 9:** Internal consistency estimates for the total LSS and its ten dimensions.

LSS Dimension	No. of Items	Cronbach’s α	McDonald’s ω
Total LSS	83	0.963	0.964
Critical Thinking	6	0.785	0.785
Creative Thinking	10	0.880	0.881
Decision-Making and Problem-Solving	12	0.908	0.907
Coping with Stress and Emotions	11	0.873	0.873
Interpersonal Relationships and Communication	7	0.843	0.843
Empathy	7	0.890	0.889
Self-Awareness	12	0.936	0.936
Self-Esteem	8	0.929	0.931
Teamwork	5	0.837	0.843
Social Responsibility	5	0.782	0.783

Note. LSS = Life Skills Scale; α = Cronbach’s alpha; ω = McDonald’s omega. The Teamwork dimension comprises negatively worded items; therefore, higher unreversed Teamwork scores indicate less favourable attitudes towards teamwork. This should be considered when interpreting reliability estimates and total LSS scores.

## Data Availability

The data presented in this study are available from the corresponding author upon reasonable request. The data are not publicly available due to ethical and privacy restrictions.

## Supplementary Materials

The following supporting information can be downloaded at: https://www.mdpi.com/article/10.3390/ejihpe16090121/s1, Table S1: Exploratory factor analysis of the LSS: Factor loadings and uniqueness values after Varimax rotation; Table S2: Descriptive statistics for the LSS items; Figure S1: Confirmatory factor analysis model of the original ten-factor LSS structure; Figure S2: Confirmatory factor analysis model of the EFA-derived eight-factor structure.

## Abbreviations

The following abbreviations are used in this manuscript:

AVE	Average variance extracted
CFA	Confirmatory factor analysis
CFI	Comparative fit index
CI	Confidence interval
DIF	Differential item functioning
DWLS	Diagonally weighted least squares
ECVI	Expected cross-validation index
EFA	Exploratory factor analysis
ESEM	Exploratory structural equation modelling
GAD-7	Generalized Anxiety Disorder-7 item scale
GFI	Goodness-of-fit index
HTMT	Heterotrait-monotrait ratio
IFI	Incremental fit index
KMO	Kaiser–Meyer–Olkin
LSS	Life Skills Scale
ML	Maximum likelihood
MLR	Robust maximum likelihood
NNFI	Non-normed fit index
PHQ-9	Patient Health Questionnaire-9 for Adolescents
PNFI	Parsimony normed fit index
RMSEA	Root mean square error of approximation
RNI	Relative noncentrality index
SRMR	Standardised root mean square residual
TLI	Tucker–Lewis index
WHO	World Health Organization
WLSMV	Weighted least squares mean and variance adjusted
