# Supplementary material for: Assessing Life Skills and Adolescent Mental Health: Cross-Cultural Adaptation and Psychometric Evaluation of the Life Skills Scale in Portugal
Source: Eur J Investig Health Psychol Educ. 2026 Aug 22;16(9):121. doi: 10.3390/ejihpe16090121 (PMC13605295; doi:10.3390/ejihpe16090121)

Supplementary Materials

Table S1. Exploratory factor analysis of the Life Skills Scale: Factor loadings and uniqueness values after Varimax rotation.

LSS item	F1	F2	F3	F4	F5	F6	F7	F8	u^2
23	0.656								0.447
19	0.648								0.480
25	0.647								0.484
18	0.642								0.503
22	0.626								0.492
6	0.625								0.536
20	0.608								0.533
3	0.603								0.575
24	0.598								0.510
28	0.586								0.502
17	0.565								0.550
4	0.554								0.600
2	0.550								0.619
21	0.548								0.545
27	0.541								0.523
7	0.511								0.634
26	0.472				0.413				0.522
9	0.455								0.583
40	0.455								0.503
5	0.454								0.712
48	0.428				0.451				0.465
47	0.402								0.509
70		0.780							0.243
67		0.760							0.301
69		0.757							0.289
66		0.745							0.284
68		0.742							0.293
73		0.684							0.340
71		0.594							0.523
61		0.564		0.493					0.308
60		0.524		0.489					0.361
72		0.475							0.594
63		0.429		0.601					0.312

LSS item	F1	F2	F3	F4	F5	F6	F7	F8	u²
57		0.421		0.554					0.377
35			0.617						0.524
31			0.615						0.442
32			0.598						0.495
39			0.584						0.534
36			0.581						0.456
38			0.538						0.515
37			0.534						0.553
33			0.518						0.691
34			0.483						0.590
30			0.456						0.735
29			0.449						0.657
56				0.703					0.287
55				0.692					0.358
54				0.643					0.448
59				0.543					0.376
64				0.472					0.559
65				0.464					0.469
62				0.435					0.472
58				0.403					0.497
81					0.772				0.361
83					0.701				0.442
52					0.602				0.498
82					0.585				0.595
79					0.561				0.606
53					0.505				0.520
80					0.483				0.680
51					0.472				0.444
49					0.440				0.393
11						0.701			0.385
15						0.672			0.420
14						0.608			0.409
13						0.579			0.470
12						0.559			0.513
16						0.543			0.507
8						0.519			0.596
10						0.468			0.702

LSS item	F1	F2	F3	F4	F5	F6	F7	F8	u^2
76							0.797		0.343
77							0.786		0.323
78							0.736		0.397
74							0.631		0.564
75							0.534		0.624
42								0.543	0.455
43								0.484	0.435
46								0.450	0.527
1									0.747
41									0.504
44									0.645
45									0.617
50									0.520

Note. LSS = Life Skills Scale; F = factor; u^2 = uniqueness. Factor loadings below .40 are suppressed. Rotation method: Varimax.

Table S2. Descriptive statistics for the LSS items.

LSS item	Min	Max	*M*	*SD*
1	1.00	5.00	3.79	1.01
2	1.00	5.00	4.06	0.84
3	1.00	5.00	4.04	0.86
4	1.00	5.00	4.16	0.82
5	1.00	5.00	3.54	1.01
6	1.00	5.00	4.05	0.83
7	1.00	5.00	4.08	0.87
8	1.00	5.00	3.78	0.94
9	1.00	5.00	4.05	0.85
10	1.00	5.00	3.74	0.98
11	1.00	5.00	3.63	0.90
12	1.00	5.00	3.77	0.89
13	1.00	5.00	3.62	0.87
14	1.00	5.00	3.55	0.89
15	1.00	5.00	3.72	0.88
16	1.00	5.00	3.67	0.93
17	1.00	5.00	4.17	0.84
18	1.00	5.00	3.92	0.87
19	1.00	5.00	3.79	0.89
20	1.00	5.00	3.83	0.90
21	1.00	5.00	3.85	0.91
22	1.00	5.00	3.73	0.89
23	1.00	5.00	4.01	0.83
24	1.00	5.00	3.81	0.91
25	1.00	5.00	3.84	0.94
26	1.00	5.00	3.82	0.95
27	1.00	5.00	3.84	0.89
28	1.00	5.00	3.80	0.88
29	1.00	5.00	3.44	1.11
30	1.00	5.00	3.31	1.17
31	1.00	5.00	3.31	1.14
32	1.00	5.00	3.23	1.09
33	1.00	5.00	3.03	1.24
34	1.00	5.00	3.62	1.00
35	1.00	5.00	3.35	1.18
36	1.00	5.00	3.00	1.20
37	1.00	5.00	3.43	1.09
38	1.00	5.00	3.38	1.02
39	1.00	5.00	2.84	1.34
40	1.00	5.00	3.63	0.86
41	1.00	5.00	3.81	0.90
42	1.00	5.00	3.62	1.07
43	1.00	5.00	3.58	0.94
44	1.00	5.00	3.70	1.09
45	1.00	5.00	3.84	0.91
46	1.00	5.00	4.04	0.88
47	1.00	5.00	4.29	0.85

48	1.00	5.00	4.16	0.86
49	1.00	5.00	4.18	0.83
50	1.00	5.00	4.14	0.87
51	1.00	5.00	4.43	0.82
52	1.00	5.00	4.16	0.96
53	1.00	5.00	4.31	0.88
54	1.00	5.00	4.13	0.96
55	1.00	5.00	3.95	1.00
56	1.00	5.00	4.04	0.92
57	1.00	5.00	3.77	1.04
58	1.00	5.00	3.50	1.06
59	1.00	5.00	3.72	0.90
60	1.00	5.00	3.80	1.05
61	1.00	5.00	3.81	0.99
62	1.00	5.00	3.82	0.92
63	1.00	5.00	3.92	0.92
64	1.00	5.00	3.71	1.11
65	1.00	5.00	4.11	0.86
66	1.00	5.00	3.64	1.07
67	1.00	5.00	3.57	1.10
68	1.00	5.00	3.36	1.17
69	1.00	5.00	3.61	1.11
70	1.00	5.00	3.84	1.03
71	1.00	5.00	4.02	1.09
72	1.00	5.00	3.92	1.05
73	1.00	5.00	3.51	1.10
74	1.00	5.00	2.80	1.24
75	1.00	5.00	2.64	1.27
76	1.00	5.00	2.30	1.21
77	1.00	5.00	2.03	1.25
78	1.00	5.00	2.33	1.25
79	1.00	5.00	3.87	1.06
80	1.00	5.00	3.53	1.21
81	1.00	5.00	3.98	1.01
82	1.00	5.00	3.66	1.11
83	1.00	5.00	4.15	0.99

Note. LSS = Life Skills Scale; Min = minimum; Max = maximum; *M* = mean; *SD* = standard deviation. Scores range from 1 to 5. Teamwork items are negatively worded; therefore, lower scores on these items indicate more favourable attitudes towards teamwork.

Figure S1. Confirmatory factor analysis model of the original ten-factor Life Skills Scale structure.

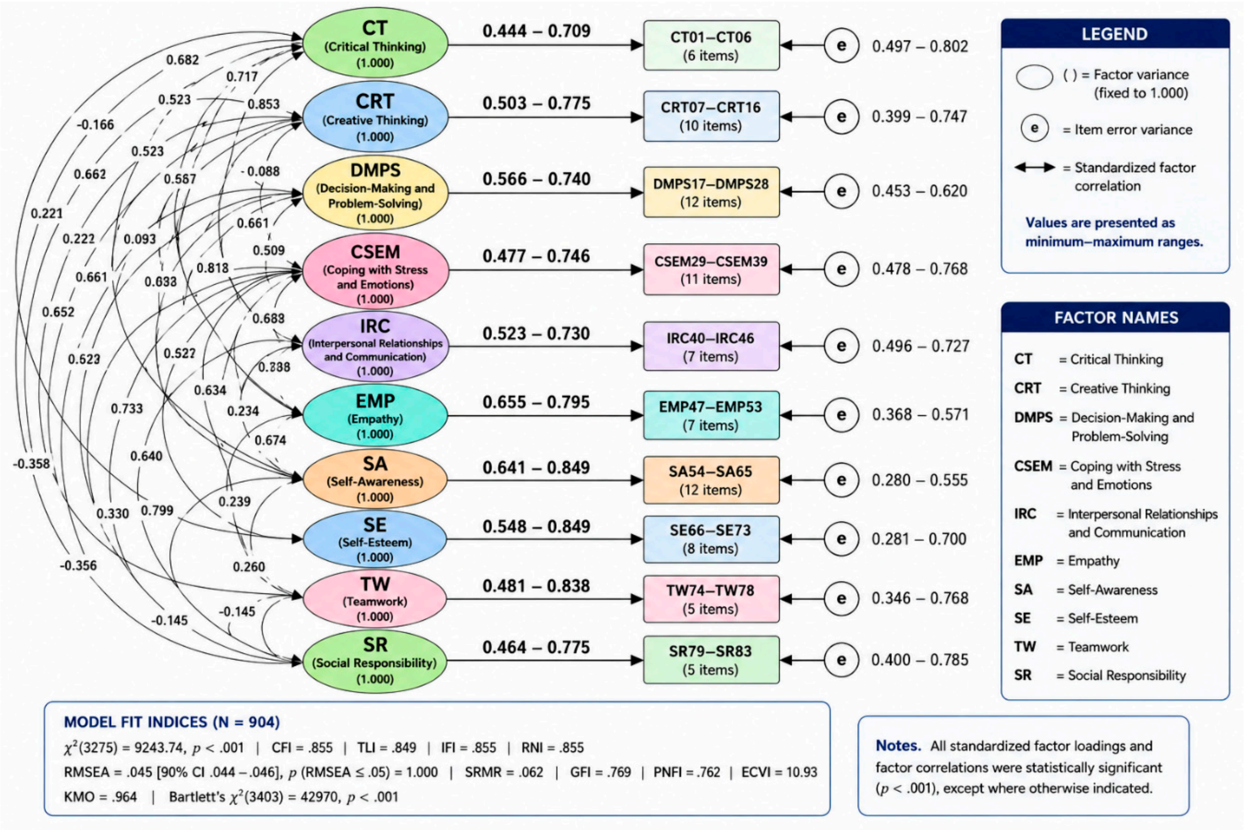

Figure S2. Confirmatory factor analysis model of the EFA-derived eight-factor structure.

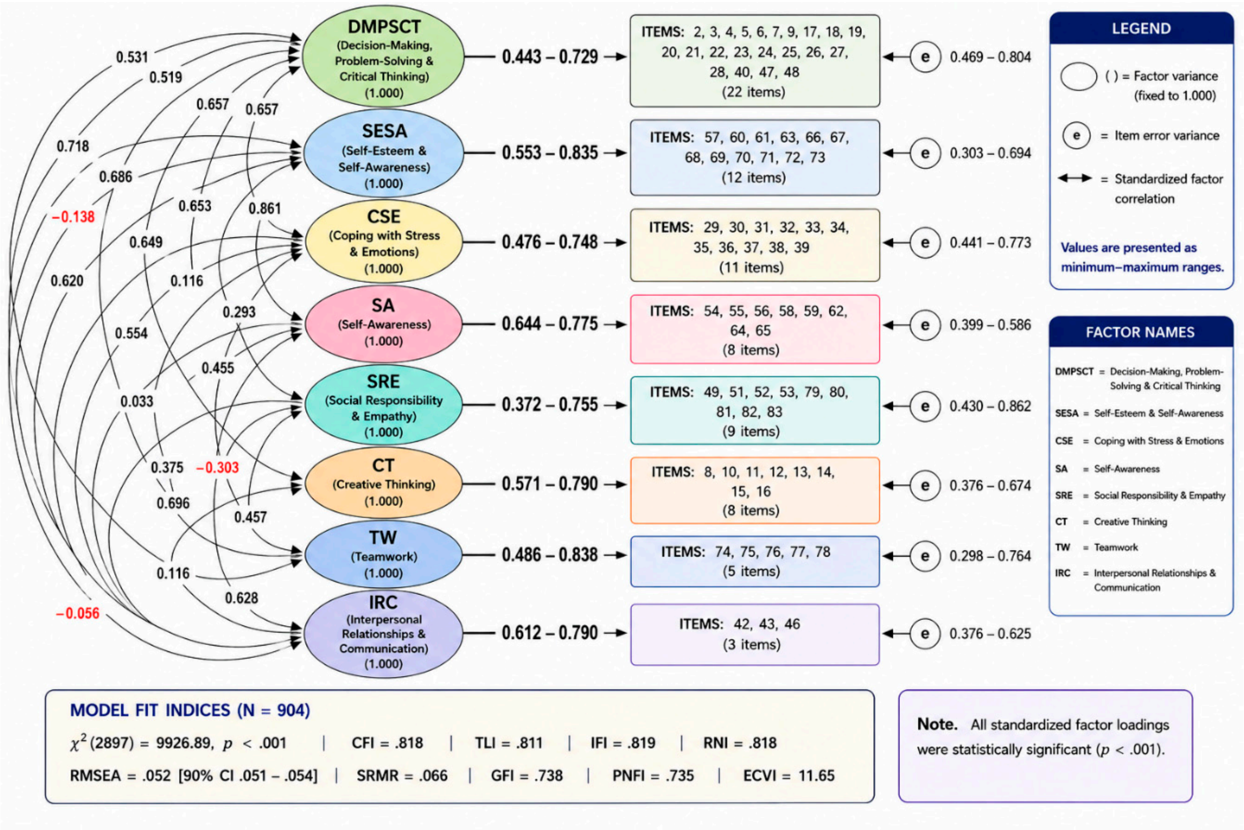

Supplement: Supplementary file 1 [file ejihpe-16-00121-s001.zip › ejihpe-4461646-supplementary.pdf]
